# Behavioral and neural evidence for an evaluative bias against other people’s mundane interracial encounters

**DOI:** 10.1093/scan/nsaa005

**Published:** 2020-01-28

**Authors:** Yin Wang, Thomas W Schubert, Susanne Quadflieg

**Affiliations:** 1 State Key Laboratory of Cognitive Neuroscience and Learning, and IDG/McGovern Institute for Brain Research, Beijing Normal University, Beijing, China; 2 Department of Psychology, University of Oslo, Oslo, Norway; 3 Instituto Universitário de Lisboa (ISCTE-IUL), Lisboa, Portugal; 4 School of Psychological Science, University of Bristol, Bristol, UK; 5 Division of Psychology, New York University Abu Dhabi, Abu Dhabi, UAE

**Keywords:** impression formation, person perception, prejudice, racial diversity, dyad perception

## Abstract

Evaluating other people’s social encounters from a third-person perspective is an ubiquitous activity of daily life. Yet little is known about how these evaluations are affected by racial bias. To overcome this empirical lacuna, two experiments were conducted. The first experiment used evaluative priming to show that both Black (*n* = 44) and White Americans (*n* = 44) assess the same mundane encounters (e.g. two people chatting) less favorably when they involve a Black and a White individual rather than two Black or two White individuals. The second experiment used functional magnetic resonance imaging to demonstrate that both Black (*n* = 46) and White Americans (*n* = 42) respond with reduced social reward processing (i.e. lower activity in the ventral striatum) and enhanced mentalizing (e.g. higher activity in the bilateral temporal–parietal junction) toward so-called cross-race relative to same-race encounters. By combining unobtrusive measures from social psychology and social neuroscience, this work demonstrates that racial bias can affect impression formation even at the level of the dyad.

## Introduction

Humans frequently draw intricate conclusions about other people’s social encounters and relations without directly getting to know them. A mere glance at two people’s non-verbal exchanges can suffice, for instance, to judge their degree of familiarity and rapport (e.g. Sekerdej *et al.,* 2018; see Quadflieg and Penton-Voak, 2017 for a review). These so-called relational first impressions often rely on well-known networks of the social brain (such as the person perception network and the mentalizing network; see Quadflieg and Koldewyn, 2017 for a review) and inform the spontaneous approval or disapproval of other people’s social conduct (e.g. Skinner and Hudac, 2017; Milinski 2016). Accordingly, many neuroscientists and psychologists consider them a fascinating mental feat as well as leap (e.g. Arioli *et al.,* 2018; Hafri *et al.,* 2018).

Alas, accumulating evidence suggests that perceivers’ rapid relational impressions of other people’s social encounters are not always accurate (Gray, 2008). In fact, far-reaching impressions concerning other people’s rapport, liking or love can be surprisingly error prone (e.g. Bernieri *et al.,* 1996; Aloni and Bernieri, 2004). Despite this realization, little is known about psychological and neural sources of inaccuracy in relational impression formation. Initial behavioral research indicates, however, that the rapid evaluation of other people’s encounters can be affected by heuristic biases that operate at the level of the dyad. Many perceivers assume, for example, that two people of dissimilar physical appearances have worse (e.g. less happy and/or less cooperative) relationships than two people of similar appearances (e.g. Forgas, 1993, 1995).

Encounters and relations between people of different racial appearances, in particular, tend to be judged less favorably than identical encounters between people of similar racial appearance by uninvolved observers (Lewandowski and Jackson, 2001). For example, the same controversial behavior (e.g. one man shoving another) is typically seen as less playful and more aggressive when it unfolds between two individuals who look racially different rather than alike (Duncan, 1976; see also Killen *et al.,* 2010). Yet, it remains uncertain whether even mundane social behaviors—such as colleagues chatting, friends partying or lovers kissing—spontaneously elicit adverse first impressions (Bhat *et al.,* 2018; Skinner and Rae, 2018). Equally unclear is which neural mechanisms may underlie this relational racial bias (Skinner and Hudac, 2017).

To address both questions, we investigated American adults’ behavioral and neural responses toward Black and White individuals who were seen to interact either within or across alleged racial boundaries. Our focus on Black–White encounters in the current work was informed by two main considerations: First, such encounters are of particular historical significance and ongoing controversy in many Western countries, including the United States of America (e.g. Craig *et al.,* 2018) and Great Britain (e.g. Eddo-Lodge, 2017). Second, initial evidence suggests that Black–White encounters (compared to other kinds of interracial encounters, see Perry and Sutton, 2006) may be particularly prone to third-party (d)evaluation as differences in people’s skin tones are particularly visible and widely considered salient markers of divergent racial identities (irrespective of whether these considerations are biologically justified; see Morning, 2017; Suzuki and von Vacano, 2018).

## Experiment 1

Experiment 1 examined Black and White Americans’ rapid evaluations of mundane social encounters between two people who looked racially different or alike using an implicit attitude test known as the evaluative priming task (EPT; Fazio and Olson, 2003).

## Method

### Participants

Native English speaking students from various institutions of higher education in New York (e.g. Columbia University, New York University) participated in in exchange for payment ($10.00) or course credit (NYU students only). A power analysis (run in G*Power) revealed that for an effect size (Cohen’s *d*) of 0.37 (based on a meta-analysis on EPT byHerring *et al.,* 2013) to be detected with 80% chance at a 5% significance level, a minimum sample of 60 participants would be required. We managed to recruit a total sample of 88 participants, including 44 participants who self-categorized as Black (23 females, age range: 18–33 years, average age: *M* = 21.75, *SD* = 4.05) and 44 participants who self-categorized as White (21 females, age range: 18–30 years, average age: *M* = 21.20, *SD* = 3.39). All participants had two biological parents of the same racial background as themselves. Written informed consent was obtained from all individuals before study participation and the study protocol was jointly approved by the Institutional Review Boards of New York University in Abu Dhabi and in New York.

### Stimuli

**Fig. 1:**
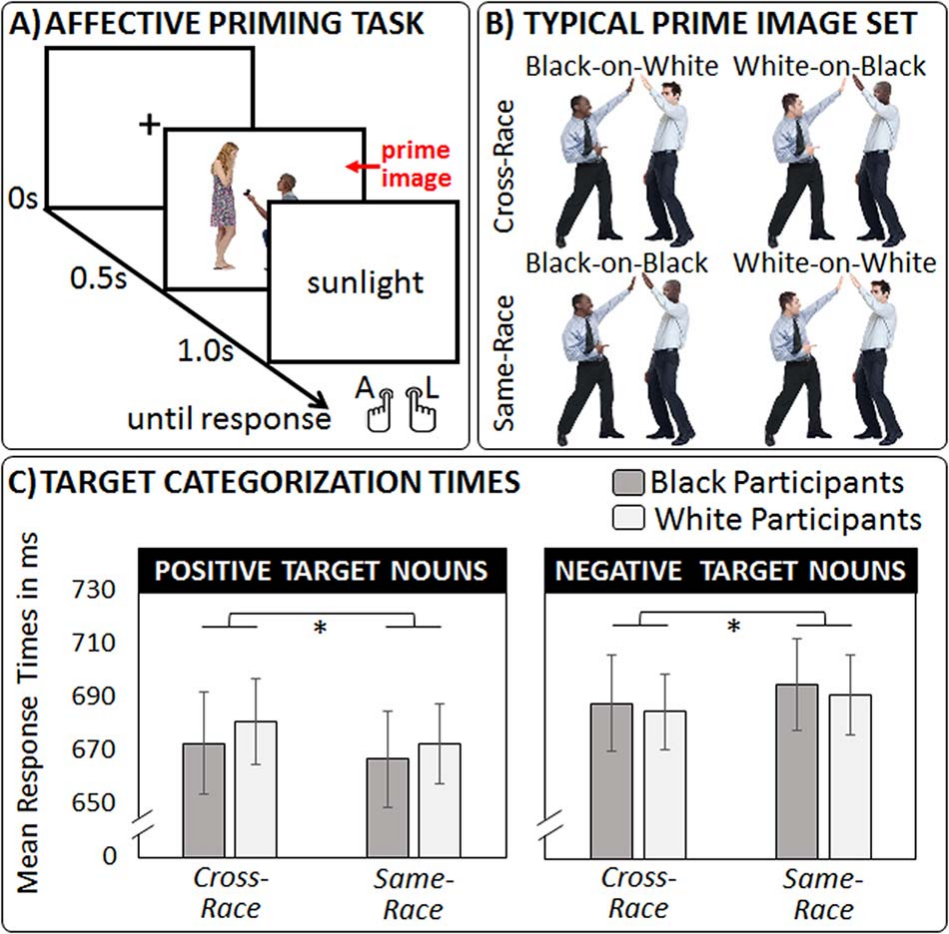
(A) In Experiment 1, participants completed a standard evaluate priming task with mundane social encounters acting as prime images. (B) Throughout the task, each encounter was displayed in four different versions to ensure that the same individuals were seen equally often in same-race and cross-race encounters. (C) Mean target categorization times were examined with a mixed measures ANOVA and revealed a significant target valence × encounter type interaction. All error bars indicate SEMS.

In the EPT (see Figure 1A), 40 positive and 40 negative (e.g. blossom *vs* avalanche) English nouns matched on length [number of letters: positive *M* = 6.75, *SD* = 1.68; negative *M* = 6.73, SD = 1.69; *t*(39) = 0.06, *P* = 0.949] and frequency [Kucera-Francis written frequency: positive *M* = 13.53, SD = 9.67; negative *M* = 12.03, SD = 11.30; *t*(39) = 0.61, *P* = 0.544] acted as target stimuli. All nouns were selected from a normed database (Bradley and Lang, 1999) and limited to low-frequency words (Chan *et al.,* 2006) that did not refer to human relationships. Prime images were 40 mundane dyadic social encounters that portrayed goal-compatible social exchanges between various interaction partners, ranging from friends (e.g. having a chat) and romantic partners (e.g. sharing a drink) to colleagues (e.g. high-fiving one another), and casual acquaintances (e.g. giving directions). None of the encounters portrayed unambiguously negative or confrontational social behaviors (e.g. interpersonal violence or competition; for further details see Figure S1 in the supplementary online material, SOM).

For each encounter, a representative color photograph was downloaded from the internet (e.g. via www.shutterstock.com). All people of relevance were then cropped out of their original background and inserted on a uniform white background. Subsequently, Adobe Photoshop© (Version 12.0.4) and additional images were used to create two types of same-race encounters [namely Black-on-Black encounters (BBE) and White-on-White encounters (WWE)] as well as two types of cross-race encounters [namely Black-on-White encounters (BWE) and White-on-Black encounters (WBE)] for each photograph (keeping all body postures and outfits constant across the four types of encounters). The appearance of allegedly White individuals was characterized by markedly lighter skin and less ‘afrocentric’ facial features than the appearance of allegedly Black individuals (see Blair *et al.,* 2004). The final set of 160 unique images was standardized to a common size (300 x 300 pixels). To avoid that idiosyncratic skin-, face- and hair-related adjustments in people’s appearances would bias our results across experimental conditions of interest, same-race encounters and cross-race encounters portrayed the exact same set of Black and White individuals, but paired them either within or between racial groups (see Figure 1B).

### Procedure and apparatus

Participants took part in a study on word comprehension in New York. Upon their arrival at the lab, they were greeted by an Asian experimenter and seated at a desk equipped with a MacBook Pro laptop (15 inch antiglare display with a screen resolution of 1280 x 1024 pixels). The EPT was presented using Cogent 2000 (University College London Functional Imaging Laboratory). Computerized instructions asked participants to view a series of image-word pairs and to categorize each word as ‘good’ or ‘bad’ as quickly and accurately as possible via a button press (i.e. A = bad, L = good). Each participant completed 320 experimental trials during which each target word was presented four times, primed once by a BBE, WWE, BWE and WBE. Trial order and prime-word pairings were randomized across participants. Following the EPT, participants also completed a brief questionnaire inquiring after their demographic background and own racial attitudes and experiences (for further details see SOM).

## Results

Participants’ mean response times on valid trials were submitted to a 2 (target valence: positive *vs* negative) × 2 (encounter type: same-race *vs* cross-race) × 2 (participant race: Black *vs* White) mixed measures analysis of variance (ANOVA; see Figure 1C). Please note that this analysis compared all same-race encounters (i.e. BBE, WWE) and all cross-race encounters (i.e. BWE, WBE), so that the exact same Black and White individuals featured in both experimental conditions (but interacted either within or across racial boundaries). Trials with erroneous replies [Blacks: *M* = 3.57%, SD = 2.55; Whites: *M* = 3.50%, SD = 2.98; *t*(86) = 0.13, *P* = 0.896, Cohen’s *d* = 0.03] and outlier replies [i.e. <150 ms or >1500 ms; Blacks: *M* = 0.94%, SD = 2.14; Whites: *M* = 1.15%, SD = 2.68; *t*(86) = 0.41, *P* = 0.682, Cohen’s *d* = 0.09] were excluded from the analysis (see Wentura and Degner, 2010).

A main effect of target valence [*F*(1,86) = 25.97, *P* < 0.001, *η*^2^*_p_* = 0.232] signaled that positive target nouns were categorized more quickly (*M* = 674, SD = 113) than negative target nouns (*M* = 689, SD = 105). This main effect was accompanied by a target valence × encounter type interaction [*F*(1,86) = 29.95, *P* < 0.001, *η*^2^*_p_* = 0.258]. Thus, the accurate classification of positive target words occurred quicker following same-race encounters (*M* = 670, SD = 110) than cross-race encounters [*M* = 677, SD = 116; *t*(87) = 3.17, *P* = 0.002, Cohen’s *d_z_* = 0.34], whereas the accurate classification of negative target words occurred quicker following cross-race encounters (*M* = 686, SD = 106) than same-race encounters [*M* = 693, SD = 106; *t*(87) = 2.71, *P* = 0.008, Cohen’s *d_z_* = 0.29]. No further main or interaction effects reached statistical significance (all *F*s < 2.56, *P* > 0.113, *η*^2^*_p_* < 0.030). Additional analyses (as presented in the SOM) confirmed that the interaction effect emerged separately for both types of same-race encounters (i.e. BBE, WWE) and that participants’ self-reported racial attitudes did not correlate with their cumulative EPT score [computed as (RT_Positive targets_Cross-race encounters_ – RT_Positive targets_Same-race encounters_) + (RT_Negative targets_Same-race encounters_ – RT_Negative targets_Cross-race encounters_) based on Herring *et al.,* 2013].

## Experiment 2

Though Experiment 1 demonstrated that mundane cross-race encounters are rapidly considered less favorably (i.e. less positive, more negative) than equivalent same-race encounters, it failed to establish which mental mechanisms underlie this bias. On the one hand, mere perceptual factors may facilitate its occurrence (see Lebrecht *et al.,* 2009). Specifically, the effortful integration of two dissimilar looking individuals into one unified scene may cause perceptual disfluency and associated feelings of negativity (Alter and Oppenheimer, 2009). Alternatively, the bias may be traced back to socio-cognitive factors. In many societies (including the USA) witnessing cross-race encounters still violates widespread expectations about who should (ideally) interact, who would (normally) interact, and/or who could (comfortably) interact (Childs, 2009). Such violations, in turn, are well known to induce feelings of negativity (e.g. Sutherland *et al.,* 2015) accompanied by effortful attributional searches to make sense of the unexpected event (e.g. Bartholow *et al.,* 2001).

To examine both possibilities, a second experiment drew on critical groundwork in social neuroscience. Recent neuroscientific studies indicate that at least two brain networks are involved in observing other people’s encounters (see Quadflieg and Koldewyn, 2017), namely the person perception network (PTN; dedicated toward their perceptual analysis) and the mentalizing network (MTN; accomplishing their socio-cognitive interpretation). An additional body of research suggests that experiencing as well as observing social events that are deemed positive (e.g. Hamilton and Meston, 2017) can elicit reward-related brain activity in areas such as the ventral striatum (VS) (e.g. Mobbs *et al.,* 2009; Fareri and Delgado, 2014). Based on these findings, we tested three hypotheses. First, we expected mundane cross-race encounters to elicit less neural activity in reward areas than equivalent same-race encounters, indicative of perceivers’ evaluative bias against them. Second, we anticipated cross-race encounters relative to same-race encounters to elicit enhanced neural activity in the PPN and/or the MTN, indicative of perceivers’ domain-specific processing efforts (see Bokde *et al.,* 2005; Koster-Hale and Saxe, 2013). Third, we predicted reduced functional connectivity between reward-related and perceptual- or mentalizing-related brain activity for mundane cross-race relative to same-race encounters, indicative of compromised relational impression formation for the former.

## Method

### Participants

As before, native English speaking students from various institutions of higher education in New York participated in exchange for payment ($50.00). One participant was excluded due to falling asleep during study completion, resulting in a final sample of 46 participants who self-categorized as Black (22 females, age range: 18–29, average age: *M* = 21.50, SD = 3.18) and 42 participants who self-categorized as White (21 females, age range: 18–29, average age: *M* = 21.00, SD = 2.88). The sample size surpassed common sample sizes in fMRI research (see Poldrack *et al.,* 2017) and was based on Experiment 1 (as we planned on re-administering the EPT). All participants had two biological parents of the same racial background as themselves, were right-handed, had no history of neurological or neuropsychiatric disorders, and took no psychoactive medication. Written informed consent was obtained from all individuals before study participation and the study’s procedure was jointly approved by the Institutional Review Boards of New York University in Abu Dhabi and New York.

### Stimuli

Participants completed three established tasks while undergoing fMRI: a categorization task (Wang and Quadflieg, 2015), a person perception task (Quadflieg *et al.,* 2011) and a mentalizing task (Dodell-Feder *et al.,* 2011). During the categorization task, the exact same set of cross-race and same-race encounters as in Experiment 1 was used (standardized to a size of 400 x 400 pixels). During the person perception task, 42 human faces (21 females), 42 human bodies (21 females) and 42 cars, as well as phase-scrambled controls for faces and bodies, were used (taken from Quadflieg *et al.,* 2011) and presented in color on a uniform gray background, standardized to a common size [184 (width) x 210 (height)]. During the mentalizing task, 20 short stories were downloaded from http://saxelab.mit.edu/superloc.php. Half of the stories described false beliefs, the other half described false photographs, signs and maps.

### Procedure

Participants were invited to take part in a study on the perception of human encounters in New York. Upon their arrival at the lab, they were greeted by an Asian experimenter and informed that the study would comprise three tasks while undergoing functional magnetic resonance imaging (fMRI): a categorization task (i.e. the main experimental task) as well as a person perception task and a mentalizing task (i.e. two so-called localizer tasks). During the categorization task, participants were asked to view images of person dyads and to indicate via a button press with their right hand whether each image displayed interpersonal helping (yes = index finger, no = middle finger). This task (inspired by Canessa *et al.,* 2012) was considered appropriate as several of our mundane encounters showed one person assisting another (e.g. carrying luggage, offering a tissue, donating money, giving directions, etc.; see Figure S1, items 05, 15, 37 and 40). The task served two main purposes: It encouraged participants to attend to both individuals per encounter and to assess all encounters on the same dimension (irrespective of their racial composition). Upon receiving six practice trials outside the fMRI scanner, participants completed the task as an event-related design inside the scanner.

In the scanner, the task comprised two separate runs, each lasting ~9 min and consisting of 80 trials (presented in a new pseudo-random order for each participant). Each of the 40 different social encounters was shown twice per run, once as a same-race and once as a cross-race encounter with different types of same-race and cross-race encounters equally distributed across both runs (i.e. resulting in 20 BBE, 20 WWE, 20 WBE and 20 BWE per run). On each trial, an encounter appeared centralized on a white screen at a visual angle of 15° x 15°. After 1500 ms, it was replaced by a black fixation cross of varying duration (between 2500 and 12 500 ms). The order of stimuli was optimized via optseq2 (http://surfer.nmr.mgh.harvard.edu/optseq/) with three different sequences being used in a counterbalanced manner across runs and participants.

Following the categorization task, the two localizer tasks were administered as previously described (see Wang and Quadflieg, 2015). In short, the person perception task required participants to view blocks of consecutively presented images (at a visual angle of 14.4 x 14.6) while performing an one-back repetition detection task. In addition, the mentalizing task required participants to read a series of short stories that either described false beliefs or false physical depictions (e.g. in photographs) before responding to a true/false statement following each story via button press. All stories and statements were presented centrally in White 40 point Arial Font against a uniform black background. In total, participants completed three runs of the person perception task and one run of the mentalizing task. All stimuli were back projected onto a screen visible via a mirror mounted on the MRI head coil. Stimulus presentation and recording of participants’ responses was accomplished using Presentation® software (Neurobehavioral Systems Inc.) and Cogent 2000 (University College London Functional Imaging Laboratory). After fMRI scanning, an abridged version of the EPT was again administered (see SOM for details).

### fMRI data acquisition, pre-processing and analysis

The fMRI session was conducted on a 3 Tesla head scanner (Siemens Allegra, Erlangen, Germany) with an eight channels array head coil. Functional images were collected using a T2*-weighted gradient EPI sequence (TR = 2000 ms, TE 30 ms, flip angle = 82°, 3 x 3 in-plane resolution; field of view 240 mm; acquisition matrix 64 x 80). For each volume, 35 axial slices parallel to the bi-commissural line with 3 mm slice thickness and 0 mm skip between slices were acquired. For each participant, 245 volumes for each run of the categorization task were collected, 267 volumes for the mentalizing localizer and 284 volumes for each run of the person perception localizer. To account for T1 saturation effects the first four volumes of each run were discarded. SPM8 software (Wellcome Department of Imaging Neuroscience, London, UK) was used for fMRI data pre-processing, which involved slice-time correction (to the middle slice of each whole-brain volume), realigning and unwarping the functional data (using a least-square approach and a six parameter rigid body spatial transformation), normalizing them to the standard EPI template, and spatially smoothing all functional data (6 mm full-width-half-maximum Gaussian kernel).

### Univariate general linear model analyses

A two-run event-related design was modeled for the categorization task that entailed a canonical hemodynamic response function (HRF) with four regressors of interest (WWE, BBE, WBE and BWE) and a 100 s high-pass temporal filter. Six motion regressors and one response time regressor were also included as nuisance regressors. For each participant, relevant contrast effect maps were computed (i.e. cross-race encounters *vs* baseline, same-race encounters *vs* baseline, cross-race *vs* same-race encounters, BBE *vs* baseline, BWE *vs* baseline, WBE *vs* baseline, WWE *vs* baseline) and then entered into second-level repeated measures ANOVAs, treating participants as a random effect. To minimize false-positive results, effects were considered statistically significant using a voxelwise threshold of *P* < 0.001, a cluster-level threshold of *P* < 0.05 (FDR corrected), and a minimum cluster size of >10 voxels. For ROI-based analyses, mean parameter estimates were extracted from the relevant trial-based contrast maps for each participant and ROI using ‘MarsBaR.’ The extracted parameter estimates were submitted to a series of 2 (encounter type: cross-race *vs* same-race) x 2 (participant race: Black *vs* White) mixed measures ANOVAs.

### Regions-of-interest localization

For the person perception task, a three-run block design was modeled, using a canonical HRF to create regressors of interest (faces, scrambled faces, bodies, scrambled bodies and cars) and a 160 s high-pass filter. Subsequently, statistical parametric maps were computed for each participant and each regressor of interest against baseline and then used to identify relevant regions of interest (ROIs). To isolate face-selective regions [i.e. the occipital face are (OFA) and the fusiform face area (FFA)], the contrast faces > cars was masked with the contrast faces > scrambled faces. To isolate body-selective regions [i.e. the extrastriate body area (EBA) and the fusiform body area (FBA)], the contrast bodies > cars was masked with the contrast bodies > scrambled bodies. For the mentalizing task, a one-run block design was modeled, using a canonical HRF to create two regressors of interest (mental states *vs* physical states) and a 128 s high-pass temporal filter. For each participant, statistical parametric maps were computed for each regressor of interest against baseline and then contrasted (i.e. mental state stories > physical state stories) to identify relevant ROIs, including the ventromedial pre-frontal cortex (VMPFC), the dorsomedial pre-frontal cortex (DMPFC), the anterior temporal lobe (aTL), the temporal–parietal junction (TPJ) and the precuneus (PrC). As in prior work (Peelen *et al.,* 2006), all ROIs were specified as a set of contiguous voxels significantly activated (*P* < 0.05, uncorrected) within a 9 mm cube surrounding a relevant peak voxel to ensure that they could be segregated from nearby activations (see SOM for peak MNI coordinates for all ROIs).

### Connectivity analyses

A psychophysiological interaction (PPI) approach was used to examine the similarity of activity patterns (‘connectivity’) between a seed region and other brain areas as a function of specific task demands (O’Reill *et al.,* 2012). The left and right VS served as seed regions in the current study. To identify both regions in each participant, significant voxels [at *P*(voxel) < 0.001, *P*(cluster) < 0.05 (FDR corrected), and a minimum cluster size of >10 voxels] in the left and right VS were determined by contrasting all experimental conditions (i.e. cross-race encounters and same-race encounters) against baseline (Quadflieg *et al.,* 2011). Applying these criteria, significant voxels were identified for 80 participants in the left VS (mean peak voxel MNI coordinates: *x* = −13, *y* = 11, *z* = −9) and for 79 participants in the right VS (mean peak voxel MNI coordinates: *x* = 16, *y* = 10, *z* = −8). Subsequently, design matrices suitable to estimate the relevant PPIs was built in SPM8 (see SOM for details) before subject-level PPI analyses were run to generate SPM contrast images which were then entered into a group-level random-effects general linear model.

### Brain-behavior correlations

To look at the relation between the strengths of participants’ cumulative score on the EPT and their neural differentiation for cross-race and same-race encounters [i.e. computed as cross-race encounters—same-race encounters], we first conducted exploratory regression analyses across the whole brain. To minimize false-positive results, effects were considered statistically significant using a voxelwise threshold of *P* < 0.001, a cluster-level threshold of *P* < 0.05 (FDR corrected), and a minimum cluster size of >10 voxels. We then looked at the relation between participants’ cumulative EPT score and their neural activity in (a) social reward processing areas as identified from the contrast same-race encounters > cross-race encounters (i.e. the left and right VS), (b) ROIs as identified from the face and body localizer and (c) ROIs as identified from the mentalizing localizer. For each ROI, we computed a difference score for the extracted mean parameter estimates (i.e. cross-race encounters–same-race encounters) and correlated these scores with participants’ cumulative EPT score.

## Results

### Evaluative priming task

As in experiment 1, the EPT produced the predicted target valence × encounter type interaction [*F*(1,86) = 8.82, *P* = 0.004, *η*^2^*_p_* = 0.093]. Follow-up pairwise comparisons revealed that the accurate classification of negative words occurred quicker following cross-race (*M* = 624, *SD* = 85) than same-race encounters [*M* = 633, *SD* = 90; *t*(87) = 2.89, *P* = 0.005, Cohen’s *d_z_* = 0.31]. For positive words, the opposite effect emerged, but failed to reach statistical significance. Thus, positive words were only slightly more quickly categorized following same-race (*M* = 606, *SD* = 82) than cross-race encounters [*M* = 610, *SD* = 89; *t*(87) = 1.56, *P* = 0.123, Cohen’s *d_z_* = 0.17]. No other effects reached statistical significance (all *F*s < 1.83, *P* > 0.179, all *η*^2^*_p_* < 0.022). As in Experiment 1, we also computed each participant’s cumulative EPT score for subsequent correlational analyses (see SOM for further details).

### Categorization task: behavioral results

During the categorization task, participants indicated that on average 42.27% (SD = 11.61) of the trials portrayed interpersonal helping. Submitting participants’ helping detection rates to a 2 (encounter type: cross-race *vs* same-race encounter) × 2 (participant race: Black *vs* White) mixed measures ANOVA returned no significant findings (all *F*s < 0.65, *p*s > 0.424, *η*^2^*_p_* < 0.009). Furthermore, participants’ detection rates did not correlate significantly with their cumulative EPT scores (all |*r*s| < 0.24; *p*s > 0.12). Additionally, participants took on average 944 ms (SD = 89) to categorize the portrayed encounters by helping. Categorization speed (after removing one unusually slow respondent, see SOM) was affected by encounter type, helping decision and participant race (all main effect *F*s > 4.89, *p*s < 0.030, *η*^2^*_p_* > 0.054; no significant interaction effects: all *F*s < 2.34, *p*s > 0.129, *η*^2^*_p_* < 0.027). Specifically, participants categorized cross-race encounters quicker than same-race encounters (*M* = 944, SD = 83 *vs M* = 952, SD = 87) and were also quicker at declaring helping absent than present (*M* = 936, SD = 89 *vs M* = 959, SD = 95). In addition, Black participants responded faster (*M* = 929 ms, SD = 79) than White participants (*M* = 967 ms, SD = 84). Mean response times on the categorization task were not correlated with participants’ cumulative EPT scores (after outlier-removal all |*r*s| < 0.19, *p*s > 0.23; see SOM).

### Categorization task: whole brain results

**Fig. 2:**
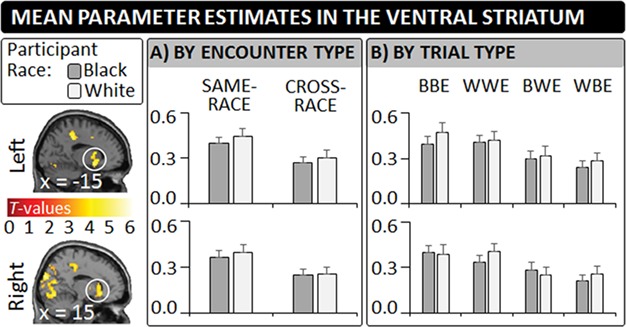
In Experiment 2, Black and White participants categorized mundane cross-race and same-race encounters according to whether they entailed helping while undergoing fMRI. An exploratory univariate whole-brain contrast revealed enhanced activity in the VS for same-race encounters relative to cross-race encounters. The bar graphs illustrate the regions’ mean parameter estimates based on participant race and (A) encounter type and (B) trial type [i.e. BBE, WWE, BWE and WBE]. All error bars indicate SEMs.

Univariate whole-brain contrasts compared participants’ neural response for same-race and cross-race encounters (Table 1). Contrasting same-race > cross-race encounters revealed increased activity in several brain regions, including the left and right VS (see Figure 2). The reverse contrast (i.e. cross-race > same-race encounters) returned enhanced activity in only three regions, specifically the DMPFC, the PrC, and the bilateral TPJ. Highly similar results were also obtained for corresponding analyses that considered participants’ race (see SOM).

**Table 1:** Peak voxel(s) in MNI coordinates and number of voxels for brain regions as identified from the categorization task by exploratory whole-brain analyses at a voxelwise threshold of *P* < 0.001, a cluster-size threshold of *P* < 0.05 (FDR corrected), and a minimum cluster size of >10 voxels

Region	Hemi-sphere	Voxels	*T*	*p*(FDR)	*x*	*y*	*z*
Same-race encounters > Cross-race encounters							
Cerebellum (extending into the fusiform gyrus)	L	146	5.22	<0.001	−24	−67	−26
			5.16	<0.001	−21	−73	−20
Cuneus	R	887	5.67	<0.001	18	−91	22
	L	78	4.56	<0.001	−18	−85	34
Inferior parietal lobule	R	59	5.21	0.002	54	−34	34
	L	56	3.80	0.002	−63	−31	28
Insula	R	87	4.95	<0.001	36	17	1
	L	123	5.23	<0.001	−33	17	7
Middle frontal gyrus	R	62	4.87	0.002	33	35	31
Posterior cingulate cortex	Midline	288	5.20	<0.001	12	−28	49
Supplementary motor area	Midline	348	6.24	<0.001	9	17	46
Ventral striatum	R	183	5.00	<0.001	15	23	−8
	L	122	5.16	<0.001	−15	11	−11
Cross-race encounters > Same-race encounters							
Dorsomedial pre-frontal cortex	Midline	339	5.82	<0.001	6	56	28
Precuneus	Midline	56	3.82	0.003	−3	−55	34
Temporal–parietal junction	R	63	4.44	0.002	54	−64	31
	L	80	3.98	0.001	−51	−67	31

### Categorization task: person perception network

Contrary to our prediction, neither of the ROIs belonging to the PPN showed a main effect of encounter type [all *F*s < 3.13, *P* > 0.080, *η*^2^*_p_* < 0.036; see SOM for further details]. Two ROIs displayed a significant encounter type × participant race interaction, namely the right FFA [*F*(1,86) = 7.04, *P* = 0.01, *η*^2^*_p_* = 0.076] and the right FBA [*F*(1,86) = 8.98, *P* = 0.004, *η*^2^*_p_* = 0.095]. These interactions signaled that Black participants showed systematically enhanced brain activity for cross-race compared to same-race encounters in both ROIs [right FFA: *t*(45) = 2.66, *P* = 0.011, Cohen’s *d*_z_ = 0.39; right FBA: *t*(45) = 3.43, *P* = 0.001, Cohen’s *d*_z_ = 0.51], whereas White participants did not [right FFA: *t*(41) = 1.15, *P* = 0.255, Cohen’s *d_z_* = 0.18; right FBA: *t*(41) = 0.95, *P* = 0.348, Cohen’s *d_z_* = 0.15].

### Categorization task: mentalizing network

**Fig. 3:**
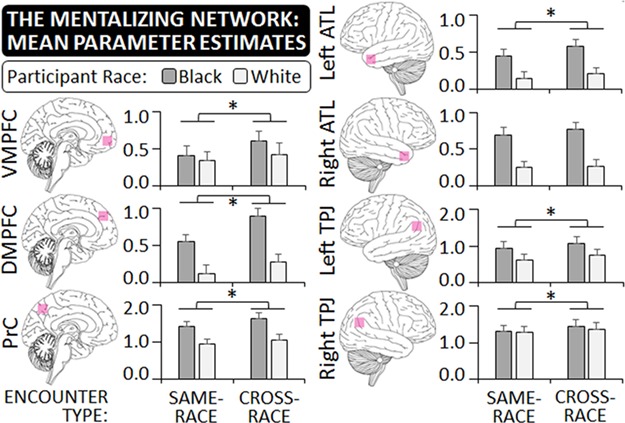
In Experiment 2, ROIs in the mentalizing network were identified with a separate localizer task and included the VMPFC, the DMPFC, the ATL, the TPJ and the PrC. The graph shows the regions’ approximate positions and mean parameter estimates based on participant race and encounter type during the categorization task. With the exception of the right ATL, all regions showed a significant activity increase when participants observed cross-race rather than same-race encounters. All error bars indicate SEMs.

With the exception of the right ATL [*F*(1,86) = 2.31, *P* = 0.132, *η*^2^*_p_* = 0.026], all ROIs of the MTN showed enhanced activity toward cross-race compared to same-race encounters (all *F*s > 6.75, *p*s < 0.012, *η*^2^*_p_* > 0.072; see Figure 3 and SOM). Participant race modulated this effect only in the DMPFC [encounter type × participant race interaction: *F*(1,86) = 4.78, *P* = 0.032, *η*^2^*_p_* = 0.053]: Here, the activity increase for cross-race compared to same-race encounters was present in both groups of participants, but was stronger in Black [*t*(45) = 6.05, *P* < 0.001, Cohen’s *d_z_* = 0.89] than White participants [*t*(41) = 2.33, *P* = 0.025, Cohen’s *d_z_* = 0.36].

### Categorization task: connectivity analyses

**Fig. 4:**
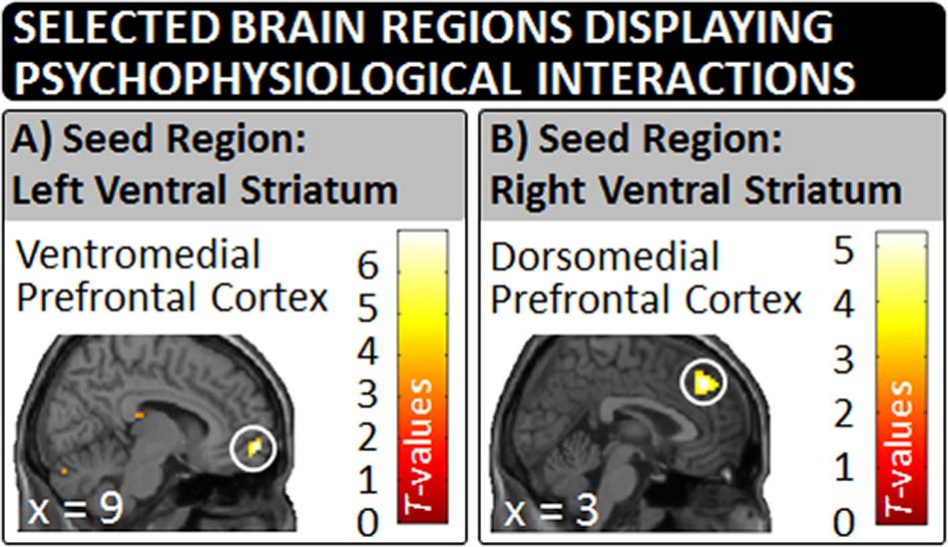
In Experiment 2, a PPI approach revealed enhanced functional connectivity during the observation of same-race relative to cross-race encounters between (A) the left VS and the VMPFC and (B) the right VS and the DMPFC.

For the left VS as the PPI’s seed region, we found enhanced functional connectivity during the observation of same-race relative to cross-race encounters with the VMPFC and the right cerebellum (Figure 4 and Table 2). For the right VS as the PPI’s seed region, we found enhanced functional connectivity during the observation of same-race relative to cross-race encounters with the DMPFC (Table 3). By contrast, we did not find enhanced functional connectivity during the observation of cross-race relative to same-race encounters between the left or right VS and other brain regions (see SOM for further details).

**Table 2:** Peak voxel(s) in MNI coordinates and number of voxels for brain regions showing systematically reduced or enhanced functional connectivity with the left VS (*n* = 80; mean MNI: *x* = −13, *y* = 11, *z* = −9) in the encounter observation task as identified by a PPI analyses in Experiment 2

Region	Hemi-sphere	Voxels	*T*	*p*(FDR)	*x*	*y*	*z*
Same-race encounters > Cross-race encounters							
Cerebellum	R	60	4.25	0.016	36	−64	−26
			3.76	0.016	27	−70	−35
Ventromedial pre-frontal cortex	Midline	58	6.97	0.016	9	59	−8
			3.83	0.016	−9	59	−8
Cross-race encounters > Same-race encounters							
No suprathreshold activation							

**Table 3:** Peak voxel in MNI coordinates and number of voxels for brain regions showing systematically reduced or enhanced functional connectivity with the right VS (*n* = 79; mean MNI: *x* = 16, *y* = 10, *z* = −8) in the encounter observation task as identified by a PPI analyses in Experiment 2

Region	Hemi-sphere	Voxels	*T*	*p*(FDR)	*x*	*y*	*z*
Same-race encounters > Cross-race encounters							
Dorsomedial pre-frontal cortex	Midline	69	5.23	0.009	3	41	40
Cross-race encounters > Same-race encounters							
No suprathreshold activation							

### Categorization task: brain-behavior correlations

**Fig. 5:**
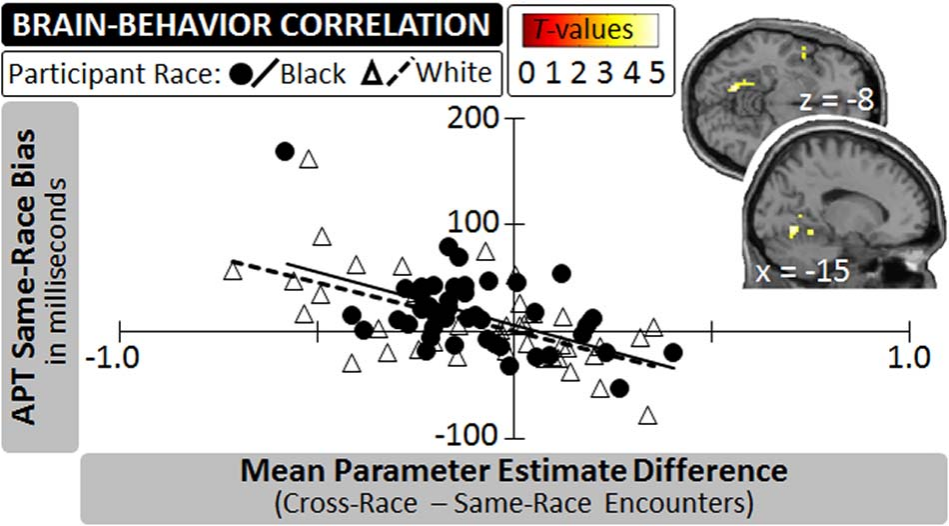
An exploratory whole-brain analysis correlating participants’ neural differentiation for cross-race and same-race encounters with their cumulative score on the EPT in Experiment 2 identified cortical activation in the left fusiform gyrus. The scatter plot illustrates this finding, displaying participants’ data by race.

An exploratory series of correlational analyses linked participants’ cumulative EPT scores to the extent of their neural differentiation between the two types of encounters (i.e. computed as cross-race encounters–same-race encounters). When adopting a whole-brain approach, this analysis returned a single suprathreshold cluster of activation which was 91 voxels in size and extended from the left medial fusiform gyrus [local peak voxel: *x* = −15, *y* = −64, *z* = −8; *t* = 4.80, *p*(FDR) = 0.001] into the cerebellum [local peak voxel: *x* = −18, *y* = −46, *z* = −14; *t* = 4.59, *p*(FDR) = 0.001]. The more this region increased its activity for same-race relative to cross-race encounters during the categorization task, the stronger was participants’ bias in favor of same-race encounters on the EPT (see Figure 5). By contrast, scrutinizing potential correlations between participants’ EPT score and their neural activity in ROIs related to reward processing (i.e. the left and right VS), mentalizing or person perception revealed no systematic associations (all |*r*s| < 0.20, all *p*s > 0.05; see SOM for details).

## Discussion

Upon witnessing social encounters from a third-person perspective, humans habitually form rapid impressions about other people’s interpersonal relations and obligations.

But it remains a matter of debate whether these relational impressions are prone to racial bias. To address this question, we measured American adults’ behavioral and neural responses to a series of mundane cross-race and same-race encounters. Using an evaluative priming paradigm, we first showed that cross-race encounters elicited less favorable evaluations than corresponding same-race encounters in both Black and White Americans (Experiments 1 and 2). This finding is noteworthy in its own right because it highlights a ‘new’ type of impression formation bias: Even though both Black and White Americans have previously been shown to respond less favorably to Black than to White individuals (for a review, see Maddox, 2004), it was not simply Black-on-Black encounters that attracted the least favorable evaluations in the current work, but rather encounters between people of different racial appearances (as hypothesized).

To better understand the bias’s underlying mental mechanisms, we then examined Black and White Americans’ neural responses toward the same-race and cross-race encounters during a basic categorization task (Experiment 2). Although participants’ categorization decisions in this task were unaffected by encounter type, their decision speed was diminished for cross-race compared to same-race encounters. This change in decision efficiency across encounter types was further accompanied by systematic neural processing differences. Specifically, we observed a marked reduction in VS activity for cross-race relative to same-race encounters. Prior research suggests that such a reduction can reflect diminished (social) reward processing (Mobbs *et al.,* 2009; Fareri and Delgado, 2014), an interpretation that would provide converging evidence for an evaluative bias against cross-race encounters. But, at this point, this conclusion rests solely on a reverse inference that requires further empirical verification (Poldrack, 2011). Future work should, thus, strive to demonstrate that race-based variations in VS activity during social encounter perception are directly associated with systematic differences in perceived encounter valence and/or desirability.

Besides differential VS activity, study 2 also revealed enhanced activity in the MTN for cross-race compared to same-race encounters. Activity in the MTN is widely believed to signal attributions that concern other people’s states, traits and/or beliefs (for a meta-analysis, see Molenberghs *et al.,* 2016) and tends to increase during the detection of ambiguous, uncommon or unexpected social events (Dungan *et al.,* 2016; Thornton *et al.,* 2019; for a review, see Koster-Hale and Saxe, 2013). Based on this work, our data suggest that perceivers’ response to cross-race encounters was characterized by increased attributional searches concerning their social content. Though this increase was ultimately associated with quicker helping decisions for cross-race relative to same-race encounters, it was also increasingly decoupled from processing in the VS. Specifically, exploratory connectivity analyses revealed that the interplay between the VS and two regions of the MTN (namely the VMPFC and DMPFC) was compromised during cross-race encounters. In other words, enhanced mentalizing in response to cross-race encounters was increasingly decoupled from their positive evaluation. This pattern of results aligns well with behavioral data showing that ambiguous social events often motivate people to mentalize harder and faster in order to overcome a compromised sense of comfort and control (Plaks *et al.,* 2005).

Little evidence was found in Experiment 2 to suggest that the evaluative bias against cross-race encounters was driven by mere perceptual disfluency. The connectivity analysis failed to uncover any brain areas beyond the MTN that changed their interplay with the VS based on encounter type. Furthermore, compared to same-race encounters, cross-race encounters elicited neither systematically increased activity in primary or secondary visual cortices, nor in brain regions dedicated toward the visual analysis of human faces and bodies. In fact, ROI-based activity increases for cross-race encounters were confined to Black participants, making them an unlikely explanatory candidate for a bias present in both Black and White participants. It was found, however, that same-race encounters elicited enhanced activity in visual processing areas (e.g. in the cuneus, left medial fusiform gyrus) and that participants’ EPT bias in favor of these encounters was stronger, the more activity in the left medial fusiform gyrus was enhanced for same-race relative to cross-race encounters. These results require future inquiry as they may signify intensified perceptual integration of same-race encounters and, thus, imply a complementary role for basic perceptual operations in the emergence of the evaluative bias (see Papeo and Abassi, 2019).

Despite these fascinating results, the current work had several limitations. First, all interpersonal encounters were exclusively portrayed via static photographs. Even though this ecologically valid approach (given the widespread use of people photography on social media) allowed us to carefully control each target’s overt behavior (thereby ruling out low-level perceptual or non-verbal confounds; see Weisbuch *et al.,* 2009) and to present high numbers of trials for both types of encounters (thereby enhancing measurement reliability; see Asendorpf *et al.,* 2013), further research is needed to understand how different methods of exposure to other people’s encounters (e.g. via dynamic videos) may affect perceivers’ behavioral and/or neural responses toward them, and ultimately the strength of their race-based evaluative bias.

Second, based on our data, the exact nature of the observed evaluative bias remains elusive. The fMRI data primarily provide evidence of weaker positive (i.e. reward-related) evaluations for cross-race relative to same-race encounters. The EPT data, by contrast, demonstrate stronger negative evaluations for cross-race relative to same-race encounters. Though weaker positive evaluations were also captured with the EPT in Experiment 1, this observation did not reach significance in Experiment 2. Procedural changes may account for this difference: Compared to Experiment 1, the EPT in Experiment 2 was shortened and administered after a lengthy fMRI session showing the exact same (prime) images. In consequence, familiarity with the (smaller set of) prime images was greater in Study 2 than in Study 1. This circumstance may not only explain why participants’ RTs on the EPT were generally faster in Study 2 than in Study 1, but also why the task may have been less sensitive to detect RT modulations based on encounter type overall. Nevertheless, forthcoming investigations will need to clarify the relative contribution of both positive and negative evaluations toward the evaluative bias against cross-race encounters.

Third, the exact socio-cognitive and/or perceptual processes that cause the evaluative bias remain to be determined. At this point, various psychological mechanisms could explain the observed neural activity in response to cross-race encounters. Such encounters may, for example, violate expectations about desirable interpersonal conduct (i.e. the expectation that members of different racial groups should not mingle; Eller *et al.,* 2017), common social conduct (i.e. the expectation that members of different racial groups do not usually mingle; Pescosolido *et al.,* 1997), and/or pleasant social conduct (i.e. the expectation that members of different racial groups are prone to discomfort when they mingle; Avery *et al.,* 2009). In addition, cross-race encounters’ complex visual features (e.g. enhanced stimulus contrast) may pose a unique perceptual challenge for third-party observers (Spillmann and Werner, 1996). Accordingly, further research is needed to identify which psychological processes give rise to the observed bias and whether they affect different groups of perceivers (e.g. Black and White participants) in the same way.

In this context, it must also be highlighted that our experiments were well powered to detect the mere arousal of evaluative bias against positive cross-race encounters in both Black and White perceivers. In contrast, our experiments were less suited to capture (potentially small to moderate) differences in the nature of this bias across Black and White perceivers (Poldrack *et al.,* 2017). Therefore, it remains to be examined whether perceivers of different racial backgrounds hold truly equivalent biases toward mundane cross-race encounters (see Skinner and Rae, 2018). With this goal in mind, future studies on the neural correlates of the observed bias should also aim to adopt designs that lend themselves well to exploring potential differences across (groups of) perceivers in a more comprehensive manner (e.g. via representational similarity analysis; Nili *et al.,* 2014).

Equally relevant for further inquiry is the question whether our findings may generalize to the perception of other forms of interracial contact. Asian-White encounters, for instance, may be less prone to third-party devaluation than Black–White encounters due to systematic differences in race-specific cultural stereotypes and/or perceived race saliency (see Lewandowski and Jackson, 2001). Finally, it remains to be addressed whether the observation of interracial crowds may prompt similar evaluative biases as the observation of interracial dyads, especially when such crowds are expectancy-incongruent (e.g. white-minority/black-majority crowds relative to white-majority/black-minority crowds in contemporary America; see Lamer *et al.,* 2018).

Despite these limitations, the obtained data make several important theoretical contributions: Using both behavioral as well as neural measures, they demonstrate that rapid race-based evaluative bias occurs in response to mundane person dyads (i.e. with people spontaneously favoring same-race dyads over cross-race dyads) and suggest that this dyadic racial bias may be fueled by both socio-cognitive and perceptual processes. These findings urge social scientists to investigate the bias’s societal consequences. On the one hand, this bias may undermine people’s ability to accurately detect and/or appreciate cross-racial rapport, liking and/or love and, thus, contribute to feelings of discomfort in racially diverse environments (Burrow and Hill, 2013). On the other hand, the bias’s accompanying mentalizing efforts may provide a unique opportunity to re-think and unlearn racial prejudice as recently postulated by vicarious contact theory (Vezzali *et al.,* 2014). In light of these possibilities, advancing our understanding of how mundane cross-race encounters are interpreted and evaluated from a third-person perspective is an endeavor of particular scientific urgency.

## Funding information

This work was made possible by a New York University|Abu Dhabi start-up grant awarded to S.Q.

## Supplementary Material

scan-19-201-File011_nsaa005

## Data Availability

The data and code supporting the findings of this study are available from the corresponding authors upon request.
