# Supplementary material for: Behavioral and neural evidence for an evaluative bias against other people’s mundane interracial encounters
Source: Soc Cogn Affect Neurosci. 2020 Jan 28;14(12):1329–39. doi: 10.1093/scan/nsaa005 (PMC7137724; doi:10.1093/scan/nsaa005)
Supplement: scan-19-201-File011_nsaa005 [file scan-19-201-file011_nsaa005.docx]

**REVIEWED SUPPLEMENTARY ONLINE MATERIAL (SOM)**

**EXPERIMENT 1: ADDITIONAL STIMULUS INFORMATION**

During the Evaluative Priming Task (EPT) participants viewed 40 mundane social encounters as shown in Figure S1 (in their cross-race version).

| 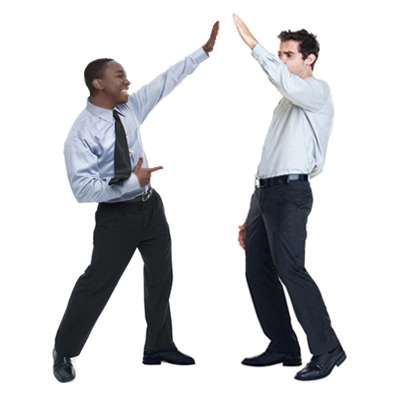01. High five | 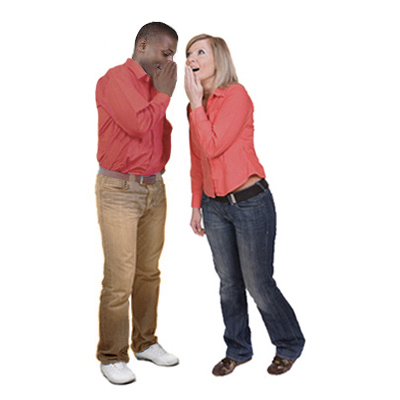02. Gossip | 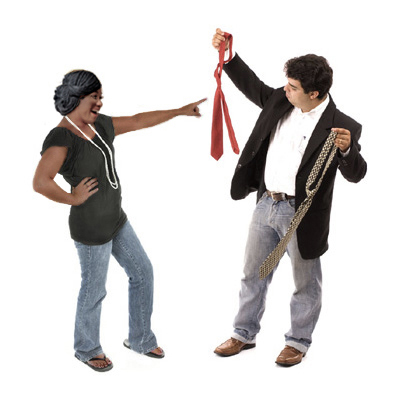03. Choice | 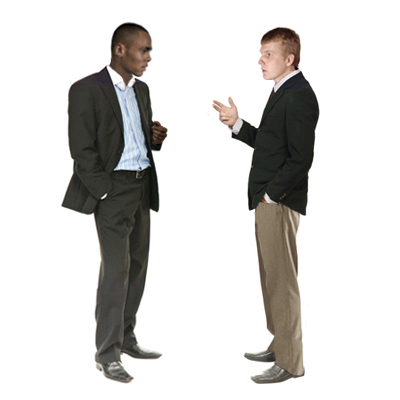04. Chat | 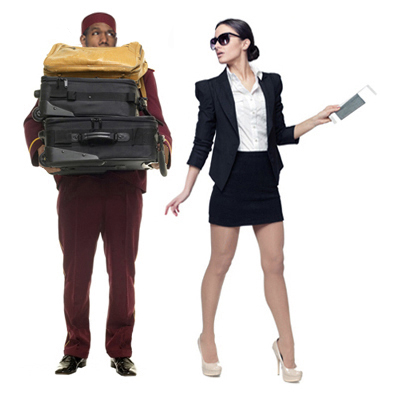  05. Luggage | 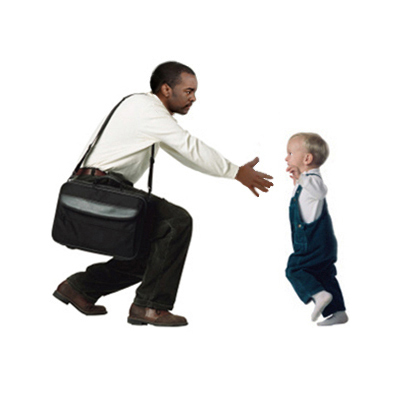06. Hug |
| --- | --- | --- | --- | --- | --- |
| 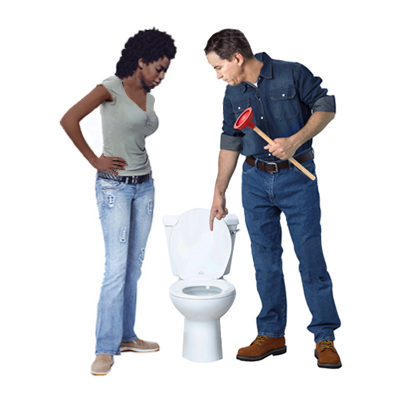07. Plumbing | 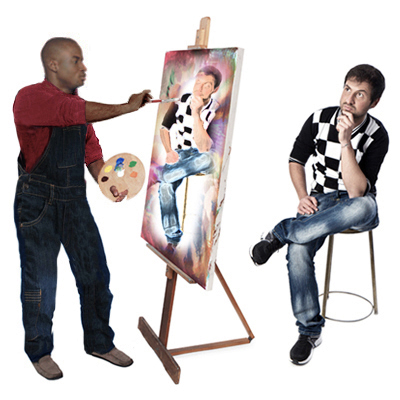08. Portrait | 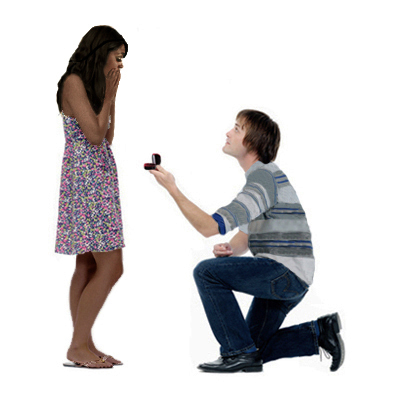 09. Proposal | 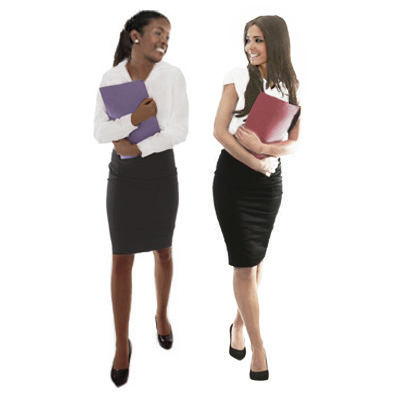10. Walk | 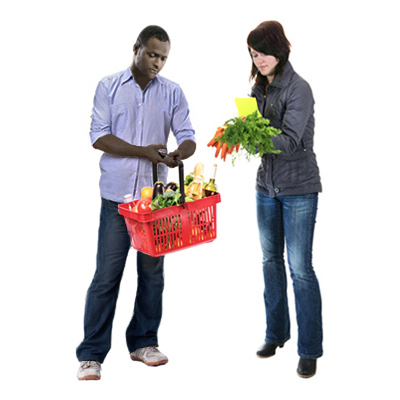11. Shopping | 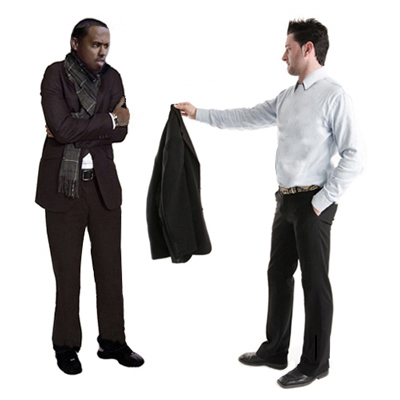12. Cold |
| 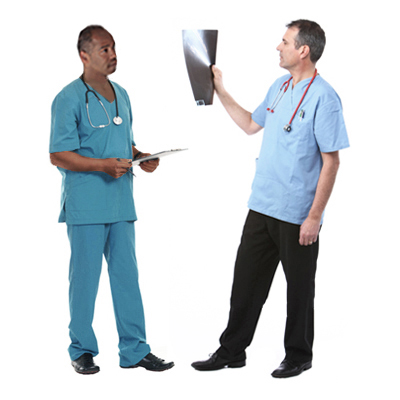 13. X-ray | 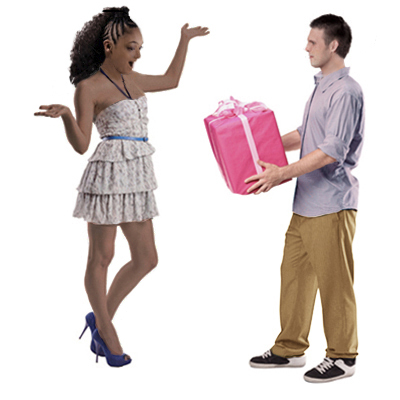14. Gift | 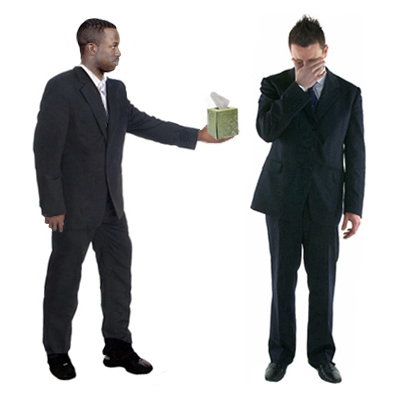15. Comfort | 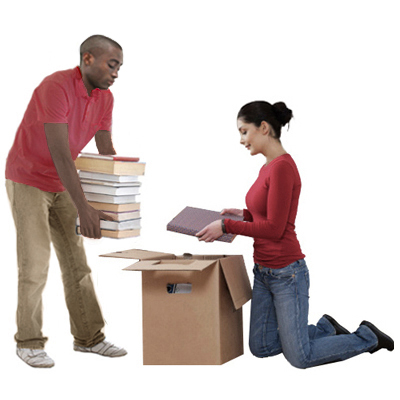16. Books | 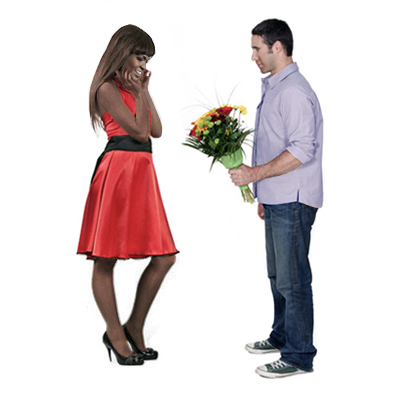 17. Flowers | 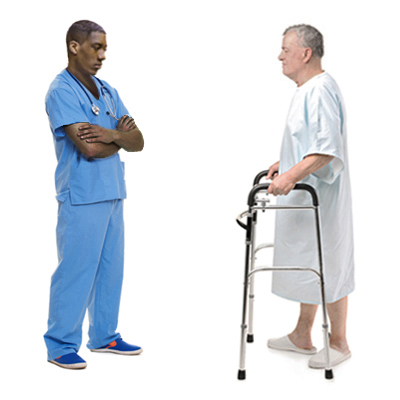 18. Patient |
| 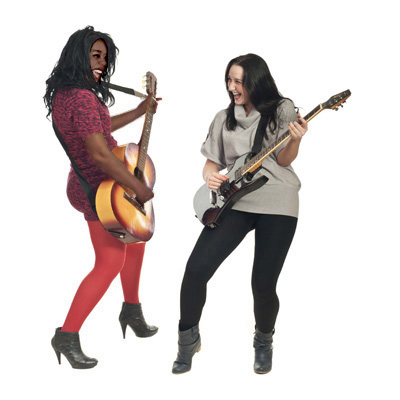19. Band | 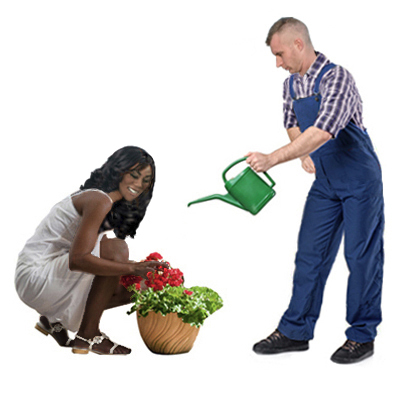20. Plant | 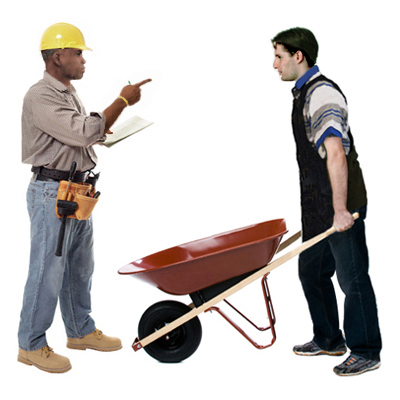 21. Work | 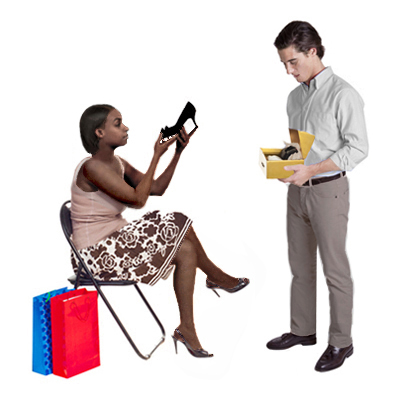22. Sale | 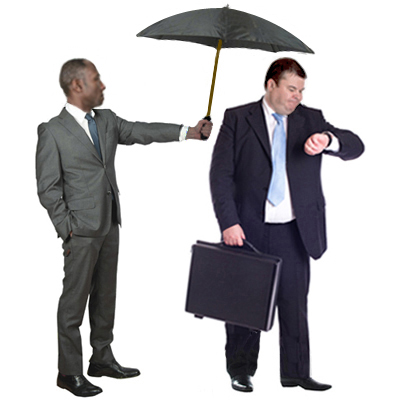23. Umbrella | 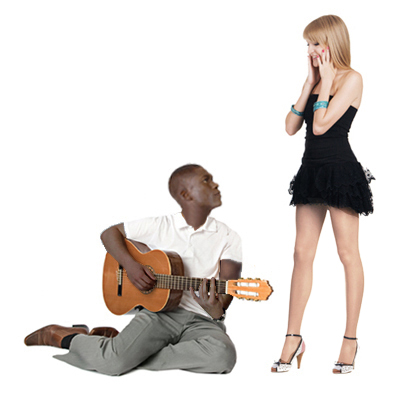 24. Song |
| 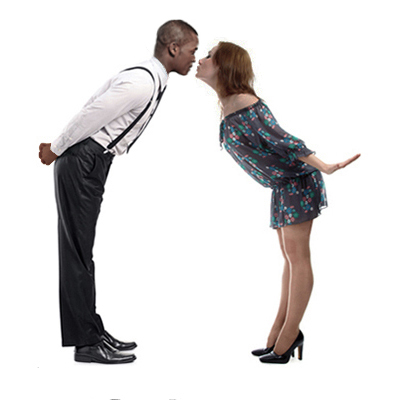 25. Kiss | 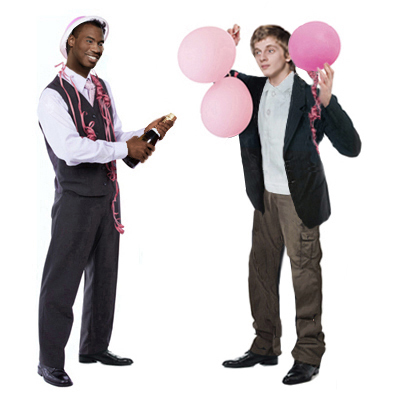26. Party | 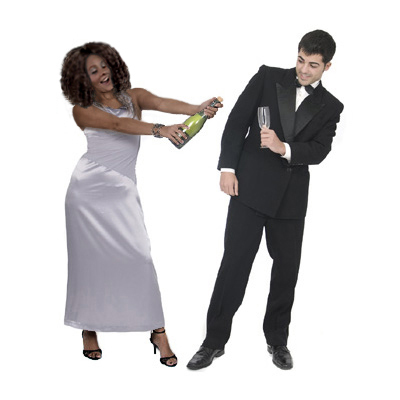 27. Cheers | 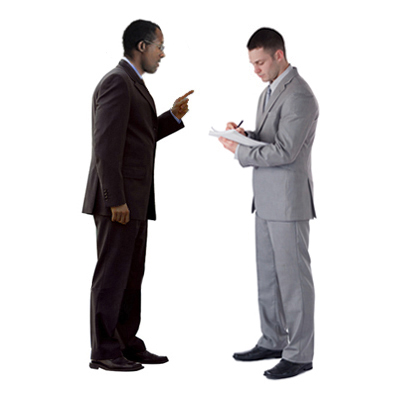28. Briefing | 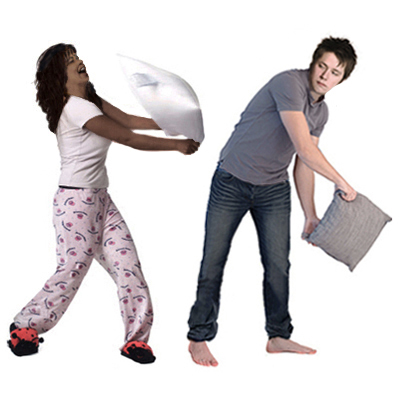29. Pillows | 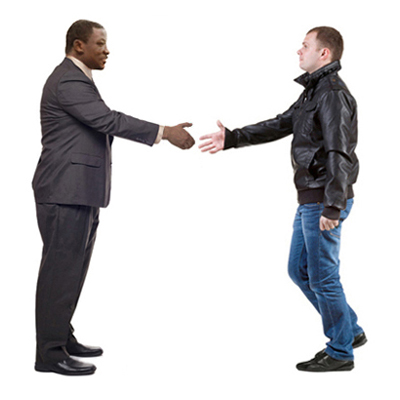30. Greeting |
| 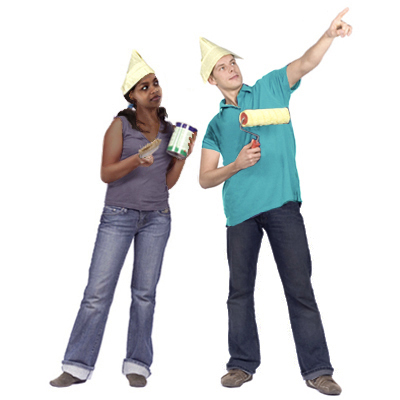31. Painting | 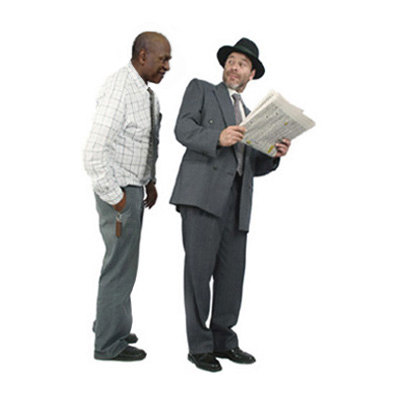32. News | 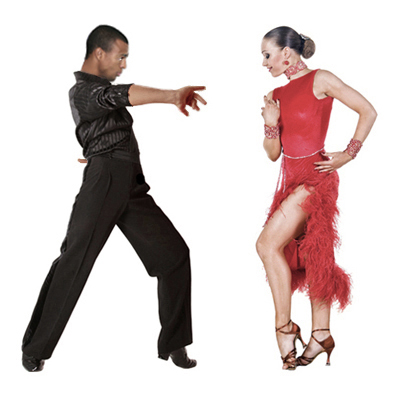 33. Dance | 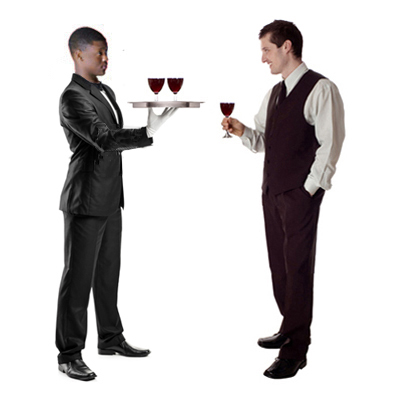 34. Drink | 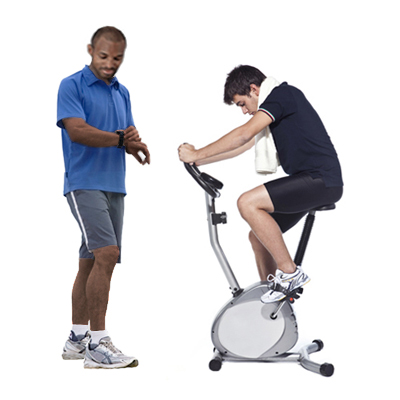35. Coach | 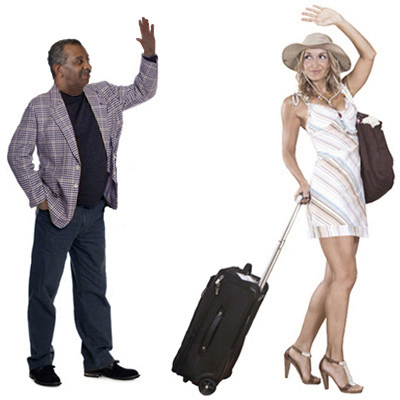36. Goodbye |
| 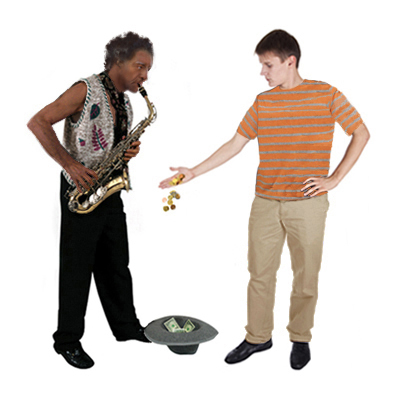37. Music | 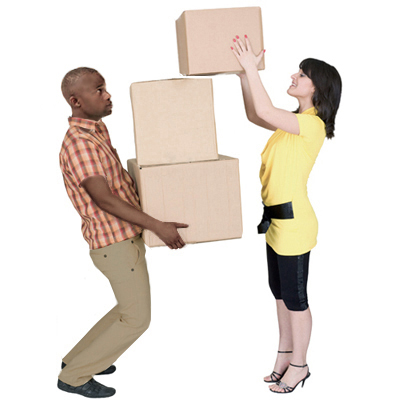  38. Boxes | 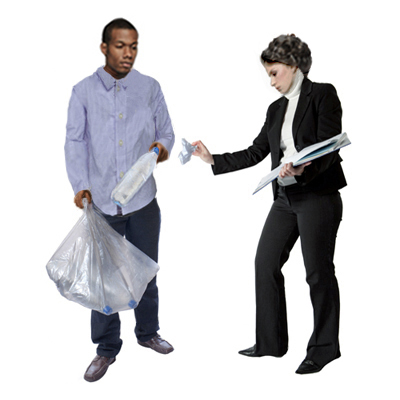  39. Waste | 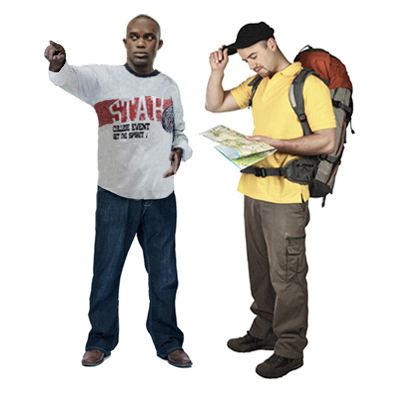  40. Directions | |  |

*Figure S1*. Thumbnails of all dyadic encounters presented in Experiment 1. Encounters were prepared by downloading photographs from www.shutterstock.com. All thumbnails are reproduced in adherence with the company’s standard license terms of service (http://www.shutterstock.com/licensing.mhtml).

**EXPERIMENT 1: ADDITIONAL PROCEDURAL DETAILS**

During the Evaluative Priming Task (EPT), participants first received a block of practice trials that entailed the 80 target nouns and 6 filler nouns. On each practice trial, a noun appeared in the center of a White screen (in 70 point Black Arial font) and participants pressed one of two keyboard buttons (i.e., A = bad, L = good) with their index fingers. If participants’ reply took longer than 2 seconds or resulted in a misclassified word, a red cross signaled that their answer was invalid. After 86 practice trials, participants were asked to categorize the exact same target words again, but this time the words followed a color image. To ensure that participants would not ignore these images, they were told that their image memory would subsequently get tested (cf. Fazio, Jackson, Dunton, & Williams, 1995). They then received 6 practice trials with image-word pairs not used in the main experiment before completing 320 experimental trials (subdivided into two blocks of 160 trials). Each trial started with a 500 ms black fixation cross in the center of a White screen. The cross was then replaced by a 500 ms prime image (at a visual angle of 13°×13°) followed by a target word (in 70 point Black Arial Font) that stayed on the screen until participants gave a response or 2000 ms elapsed. The intertrial interval was 1000 ms. Following the EPT, participants completed a brief questionnaire. After reporting their sex (male or female), race (Black, White, or Other), and age (in years), participants were asked to rate the intensity of their exposure to cross-race encounters (based on Islam & Hewstone, 1993) using a 7-point scale (from 1=*none at all* to 7=*very much*) in response to five separate items, such as “In everyday life, how often do you witness interactions between Blacks and Whites in your neighborhood?”. Then they completed a two-item race saliency measure (Voci & Hewstone, 2003). The stem statement “When meeting a person for the first time…” was accompanied by “I pay attention to his/her race” and “I do not care about her/his race” (reverse-coded). Replies were again given on 7-point scale (with 1=*strongly disagree* to 7=*strongly agree*). Using the same 7-point scale, participants subsequently completed a four-item similarity measure (including items such as “Blacks and Whites in the U.S. hold similar goals in life”; Leach et al., 2008), a ten-item race conception measure (including items such as “Racial groups are primarily identified by biology”; Williams & Eberhardt, 2008), and a six-item racial preference scale (including items such as “I would prefer to live in a neighborhood with people of my own racial origin”; Wolsko, Park, & Judd, 2006). Finally, participants completed the Short Social Dominance Orientation scale (Pratto et al., 2013) by indicating how much they supported statements such as “In setting priorities we must consider all racial groups” (reverse coded) on a 10-point scale (1 = *extremely oppose* to 10 = *extremely favor*).

**EXPERIMENT 1: ADDITIONAL RESULTS**

*Evaluative Priming Task:* It was examined whether the observed interaction effect emerged for both types of same-race encounters (see Table S1), regardless whether they portrayed racial minority members (i.e., BBE) or racial majority members (i.e., WWE). Separate analyses confirmed that significant target valence × encounter type interactions as described above were found for the relevant 2 (target valence: positive vs. negative) × 2 (encounter type: BBE vs. cross-race) × 2 (participant race: Black vs. White) mixed measures ANOVA [*F*(1,86) = 8.70, *p* = .004, *η*^2^*_p_* = .092] as well as for the complementary 2 (target valence: positive vs. negative) × 2 (encounter type: WWE vs. cross-race) × 2 (participant race: Black vs. White) mixed measures ANOVA [*F*(1,86) = 18.78, *p* < .001, *η*^2^*_p_* = .179]. Neither of these two-way interactions was qualified by an additional three way interaction (both *F*s < 0.83, *p* > .366, *η*^2^*_p_* < .010). We then determined participants’ cumulative EPT score. It ranged from -37.44 to 72.72 (*M* = 13.11, *SD* = 22.34) with larger values signalling a stronger preference for same-race over cross-race encounters.

*Questionnaire Results:* Participants’ average replies on the self-report measures revealed that they often witnessed cross-race encounters in their own lives and generally held egalitarian racial attitudes (see Table S2). For instance, prompted to rate how often they witnessed other people’s cross-race encounters, participants reported an average exposure above the 7-point scale’s midpoint [*M* = 4.87, *SD* = 1.07, *t*(87) = 7.65, *p* < .001, Cohen’s *d* = 0.82]. Furthermore, asked whether they preferred to mingle with people of their own race, they refuted the idea with an average rating below the 7-point scale’s midpoint [*M* = 2.46, *SD* = 1.03, *t*(87) = 13.99, *p* < .001, Cohen’s *d* = 1.49]. But at the adequate Bonferroni-corrected statistical threshold for the six scales (i.e., *p* = .008), no systematic correlations between participants’ questionnaire scores and their cumulative EPT score emerged (see Table S3).

Table S1. *Mean response times (including standard deviations) on the evaluative priming task based on target valence, trial type, and participant race in Experiment 1*

| Target Valence | Prime Image | Black Sample | White Sample | *t*-value (df = 86) | *p*-value | Cohen’s *d* |
| --- | --- | --- | --- | --- | --- | --- |
| Negative | BBE | 696 (113) | 688 (100) | 0.34 | .737 | 0.07 |
|  | WWE | 693 (120) | 693 (98) | 0.02 | .988 | 0.00 |
|  | BWE | 688 (114) | 684 (97) | 0.21 | .834 | 0.04 |
|  | WBE | 687 (122) | 686 (96) | 0.04 | .969 | 0.01 |
| Positive | BBE | 669 (119) | 677 (101) | 0.36 | .718 | 0.07 |
|  | WWE | 666 (120) | 669 (108) | 0.13 | .899 | 0.03 |
|  | BWE | 672 (126) | 677 (104) | 0.22 | .830 | 0.04 |
|  | WBE | 675 (128) | 684 (110) | 0.37 | .712 | 0.08 |

Abbreviations: Black-on-Black encounters (BBE), White-on-White encounters (WWE), Black-on-White encounters (BWE), White-on-Black encounters (WBE).

Table S2. *Participants’ race-related attitudes and experiences as measured in Experiment 1*

| Measure | Cronbach’s Alphas | | Means and Standard Deviations | | Subsample Comparison | | |
| --- | --- | --- | --- | --- | --- | --- | --- |
|  | Black Sample | White Sample | Black Sample | White Sample | *t*-value | *p*- value | Cohen’s *d* |
| Contact exposure | .69 | .74 | 4.80 (1.10) | 4.95 (1.05) | 0.69 | .493 | 0.14 |
| Race saliency | .50 | .27 | 3.52 (1.60) | 3.25 (1.19) | 0.91 | .368 | 0.19 |
| Race similarity | .55 | .68 | 4.20 (1.00) | 5.50 (0.93) | 6.32 | <.001* | 1.36 |
| Race conception | .68 | .79 | 4.02 (0.91) | 4.11 (0.97) | 0.51 | .611 | 0.10 |
| Race preference | .76 | .79 | 2.46 (0.98) | 2.47 (1.09) | 0.02 | .984 | 0.01 |
| Social dominance | .43 | .51 | 1.78 (0.91) | 1.82 (0.91) | 0.21 | .838 | 0.04 |

*Denotes *p*-values < 0.05 (Bonferroni-corrected for 6 tests).

Table S3. *Correlations (with p-values) between participants’ cumulative evaluative priming task score (EPT score) and their self-reported racial attitudes in Experiment 1*

| Scale | Black Sample | White Sample | Total Sample |
| --- | --- | --- | --- |
| Contact exposure | -.02 (.880) | -.08 (.623) | -.05 (.646) |
| Race saliency | .05 (.753) | .31 (.041) | .16 (.132) |
| Race similarity | -.03 (.831) | -.16 (.298) | -.08 (.489) |
| Race conception | -.28 (.069) | -.25 (.105) | -.26 (.014) |
| Race preference | -.10 (.501) | -.16 (.291) | -.14 (.205) |
| Social dominance | -.11 (.489) | -.01 (.936) | -.06 (.598) |

*Denotes *p*-values < 0.05 (Bonferroni-corrected for 6 tests per sample).

**EXPERIMENT 2: ADDITIONAL PROCEDURAL DETAILS**

*Evaluative Priming Task:* Following the lengthy fMRI session in Experiment 2, the EPT was shortened to avoid participant fatigue. A subset of 20 original encounters from Experiment 1 were chosen to act as prime stimuli and a subset of 20 positive and 20 negative words to act as target stimuli. As before, both sets of target words were matched on length [number of letters: positive *M* = 7.10, *SD* = 1.62; negative *M* = 7.10, *SD* = 1.62; *t*(19) = 0.00, *p* < .001] and frequency [Kucera-Francis written frequency: positive *M* = 10.25, *SD* = 9.45; negative *M* = 10.70, *SD* = 7.28; *t*(19) = 0.18, *p* = .860]. The task’s overall procedure remained unchanged: Participants were again familiarized with all target words in a block of practice trials (including 40 target words, 6 practice words). They then received 6 practice trials in which they saw image-word pairs in preparation for the final block of 160 trials.

*Region-of-Interest (ROI) Localization:* Tables S4 and S5 list the average coordinates of ROIs in the two networks (PPN and MTN) for Black and White participants.

*Connectivity Analyses:* For each PPI analysis, we created a new general linear model (GLM) with (i) a *physiological* regressor in which the seed region’s time course (i.e., first eigenvariate) was deconvolved to estimate the underlying neural activity; (ii) a *psychological* regressor in which encounter type trials (vs. baseline trials) were convolved with the canonical hemodynamic response function; and (iii) a *PPI interaction* regressor in which the psychological regressor was multiplied by the physiological regressor. We used this interaction regressor to identify voxels in which functional activity covaried in a task-dependent manner with the seed region.

Table S4. *Mean MNI coordinates of brain regions constituting the person perception network as determined by an independent localizer task in Experiment 2*

| Region | Hemisphere | *N* | *x* | *y* | *z* |
| --- | --- | --- | --- | --- | --- |
| *Black Sample (n = 46)* | | | | | |
| Occipital Face Area | R | 46 | 44 | -72 | -16 |
|  | L | 44 | -42 | -73 | -16 |
| Fusiform Face Area | R | 46 | 44 | -47 | -25 |
|  | L | 45 | -43 | -48 | -24 |
| Extrastriate Body Area | R | 46 | 50 | -73 | -3 |
|  | L | 46 | -48 | -75 | -1 |
| Fusiform Body Area | R | 46 | 45 | -48 | -25 |
|  | L | 43 | -44 | -47 | -25 |
| *White Sample (n = 42)* | | | | | |
| Occipital Face Area | R | 42 | 44 | -76 | -15 |
|  | L | 39 | -42 | -76 | -14 |
| Fusiform Face Area | R | 42 | 43 | -49 | -26 |
|  | L | 40 | -43 | -49 | -25 |
| Extrastriate Body Area | R | 42 | 47 | -75 | 0 |
|  | L | 42 | -46 | -78 | 2 |
| Fusiform Body Area | R | 42 | 45 | -49 | -24 |
|  | L | 42 | -44 | -50 | -23 |

Table S5. *Mean MNI coordinates of brain regions constituting the mentalizing network as determined by an independent localizer task in Experiment 2*

| Region | Hemisphere | *N* | *x* | *y* | *z* |
| --- | --- | --- | --- | --- | --- |
| *Black Sample (n = 46)* | | | | | |
| Ventromedial Prefrontal Cortex | Midline | 46 | 1 | 56 | -9 |
| Dorsomedial Prefrontal Cortex | Midline | 46 | -2 | 56 | 34 |
| Anterior Temporal Lobe | R | 46 | 53 | 0 | -32 |
|  | L | 46 | -53 | -1 | -31 |
| Temporal-Parietal Junction | R | 46 | 53 | -58 | 26 |
|  | L | 46 | -50 | -60 | 29 |
| Precuneus | Midline | 46 | 1 | -58 | 40 |
| *White Sample (n = 42)* | | | | | |
| Ventromedial Prefrontal Cortex | Midline | 42 | 1 | 56 | -10 |
| Dorsomedial Prefrontal Cortex | Midline | 42 | 3 | 56 | 32 |
| Anterior Temporal Lobe | R | 42 | 53 | 1 | -32 |
|  | L | 41 | -53 | 0 | -32 |
| Temporal-Parietal Junction | R | 42 | 53 | -55 | 23 |
|  | L | 42 | -52 | -60 | 25 |
| Precuneus | Midline | 42 | 1 | -57 | 40 |

**EXPERIMENT 2: ADDITIONAL RESULTS**

*Evaluative Priming Task:* As in Experiment 1, trials that resulted in errors [Blacks: *M* = 5.59%, *SD* = 4.00; Whites: *M* = 4.62%, *SD* = 3.11; *t*(86) = 1.26, *p* = .210, Cohen’s *d* = 0.27] or extremely quick/slow replies [Blacks: *M* = 0.54%, *SD* = 1.87; Whites: *M* = 0.40%, *SD* = 0.73; *t*(86) = 0.46, *p* = .648, Cohen’s *d* = 0.10] were excluded before participants’ mean raction times were submitted to a 2 (target valence: positive vs. negative) × 2 (encounter type: same-race vs. cross-race) × 2 (participant race: Black vs. White) mixed measures ANOVA (see Table S6). Besides the target valence × encounter type interaction effect (as reported in the main manuscript), this ANOVA returned again (as in Experiment 1) a main effect of target valence [*F*(1,86) = 36.12, *p* < .001, *η*^2^*_p_* = .296], signaling quicker response times for positive (*M* = 608, *SD* = 85) than negative target nouns (*M* = 628, *SD* = 86). No other effects reached statistical significance (all *F*s < 1.83, *p* > .179, all *η*^2^*_p_* < .022). We then computed each participant’s cumulative EPT score for subsequent correlational analyses. This time, the score ranged from -78.25 to 167.46 (*M* = 12.49, *SD* = 38.83).

Table S6. *Mean response times (and standard deviations) on the evaluative priming task based on target valence and (A) encounter type or (B) trial type in Experiment 2*

| Target Valence | Prime Image | Black Sample | | White Sample | *t*-value (df = 86) | *p*-value | Cohen’s *d* |  |
| --- | --- | --- | --- | --- | --- | --- | --- | --- |
| *By Encounter Type* | | | | | | | | |
| Negative | Same-race | 630 (91) | | 635 (89) | 0.26 | .796 | 0.06 |  |
|  | Cross-race | 619 (91) | | 629 (80) | 0.56 | .581 | 0.12 |  |
| Positive | Same-race | 601 (87) | | 611 (77) | 0.53 | .596 | 0.11 |  |
|  | Cross-race | 607 (93) | | 613 (85) | 0.34 | .737 | 0.07 |  |
| *By Trial Type* | | | | | | | | |
| Negative | BBE | 632 (91) | 638 (99) | | 0.30 | .767 | 0.06 | |
|  | WWE | 629 (96) | 632 (82) | | 0.16 | .873 | 0.03 | |
|  | BWE | 618 (88) | 633 (86) | | 0.81 | .422 | 0.17 | |
|  | WBE | 621 (98) | 626 (81) | | 0.30 | .768 | 0.06 | |
| Positive | BBE | 602 (88) | 616 (79) | | 0.78 | .439 | 0.17 | |
|  | WWE | 600 (89) | 606 (80) | | 0.29 | .775 | 0.06 | |
|  | BWE | 609 (96) | 614 (88) | | 0.25 | .806 | 0.05 | |
|  | WBE | 604 (91) | 613 (86) | | 0.46 | .649 | 0.10 | |

Abbreviations: Black-on-Black encounters (BBE), White-on-White encounters (WWE), Black-on-White encounters (BWE), White-on-Black encounters (WBE).

*Categorization Task: Helping Detection Rates.* Button presses declaring helping present or absent were logged equally often by Black (*M* = 99.98%, *SD* = .02) and White participants [*M* = 99.98%, *SD* = .01; *t*(86) = 0.13, *p* = .898, Cohen’s *d* = 0.00]. Submitting participants’ helping detection rates (see Table S7) to a 2 (encounter type: cross-race vs. same-race encounter) × 2 (participant race: Black vs. White) mixed measures ANOVA revealed no significant effects [encounter type: *F*(1,86) = 0.43, *p* = .516, *η*^2^*_p_* = .005; participant race: *F*(1,86) = 0.42, *p* = .521, *η*^2^*_p_* = .005; encounter type by participant race: *F*(1,86) = .05, *p* = .831, *η*^2^*_p_* = .001]. Yet, we noted that two participants (i.e., one Black female, one White male) declared helping present at an unusually high rate (i.e., > 3 *SD*s of the sample’s mean). Alas, removing both individuals from the analysis returned an equivalent non-significant pattern of results [encounter type: *F*(1,84) = 0.34, *p* = .564, *η*^2^*_p_* = .004; participant race: *F*(1,84) = 0.64, *p* = .425, *η*^2^*_p_* = .008; encounter type by participant race: *F*(1,84) = .13, *p* = .721, *η*^2^*_p_* = .002]. Additional correlational analyses demonstrated that participants’ helping detection rates were unrelated to their cumulative EPT score (see Table S8).

Table S7. *Helping detection rates in percentages (and standard deviations) for the full sample of participants and an outlier-removed sample of participants on the categorization task in Experiment 2*

| Sample | Prime Image | Black Sample | White Sample | *t*- value | *p*- value | Cohen’s *d* |  |
| --- | --- | --- | --- | --- | --- | --- | --- |
| *By Encounter Type* | | | | | | | |
| Full  (n = 88) | Same-race | 43.21 (12.46) | 41.52 (10.98) | 0.67 | .504 | 0.15 |  |
|  | Cross-race | 42.85 (12.80) | 41.34 (10.67) | 0.60 | .551 | 0.13 |  |
| Removed  (n = 86) | Same-race | 42.17 (10.39) | 40.40 (8.34) | 0.87 | .389 | 0.19 |  |
|  | Cross-race | 41.78 (10.64) | 40.30 (8.41) | 0.71 | .481 | 0.16 |  |
| *By Trial Type* | | | | | | | |
| Full  (n = 88) | BBE | 43.04 (12.72) | 41.13 (11.26) | 0.74 | .459 | 0.16 | |
|  | WWE | 43.37 (12.74) | 41.90 (11.30) | 0.57 | .571 | 0.12 | |
|  | BWE | 42.34 (13.10) | 40.48 (10.81) | 0.72 | .472 | 0.16 | |
|  | WBE | 43.37 (13.16) | 42.20 (10.99) | 0.45 | .654 | 0.10 | |
| Removed  (n = 86) | BBE | 42.00 (10.69) | 40.06 (8.99) | 0.91 | .368 | 0.20 | |
|  | WWE | 42.33 (10.74) | 40.73 (8.46) | 0.76 | .448 | 0.17 | |
|  | BWE | 41.17 (10.53) | 39.45 (8.63) | 0.82 | .414 | 0.18 | |
|  | WBE | 42.39 (11.48) | 41.16 (8.77) | 0.55 | .581 | 0.12 | |

Abbreviations: Black-on-Black encounters (BBE), White-on-White encounters (WWE), Black-on-White encounters (BWE), White-on-Black encounters (WBE).

Table S8. *Correlations (with p-values) between participants’ helping detection rates by on the categorization task and their cumulative evaluative priming task score (EPT score) for the full sample of participants and an outlier-removed sample of participants in Experiment 2*

| Sample | Encounter Type | Black Sample | White Sample | Total Sample |
| --- | --- | --- | --- | --- |
| Full  (n = 88) | Same-race | -.04 (.805) | .00 (.992) | -.01 (.924) |
|  | Cross-race | -.04 (.804) | -.02 (.886) | -.02 (.831) |
| Removed  (n = 86) | Same-race | -.23 (.125) | .08 (.627) | -.08 (.491) |
|  | Cross-race | -.23 (.121) | .04 (.804) | -.10 (.377) |

*Categorization Task: Response Times.* Submitting participants’ mean response times (see Table S9) to a 2 (helping decision: helping present vs. absent) × 2 (encounter type: same-race vs. cross-race) × 2 (participant race: Black vs. White) mixed measures ANOVA revealed a significant main effect of helping decision [*F*(1,86) = 8.31, *p* = .005, *η*^2^*_p_* = .088] and a significant main effect of encounter type [*F*(1,86) = 5.29, *p* = .024, *η*^2^*_p_* = .058], but no main effect of race [*F*(1,86) = 2.76, *p* = .100, *η*^2^*_p_* = .031] and no interaction effects [helping decision by participant race: *F*(1,86) = 0.00, *p* = .977, *η*^2^*_p_* < .001; encounter type by participant race: *F*(1,86) = 0.97, *p* = .326, *η*^2^*_p_* = .011; helping decision by encounter type: *F*(1,86) = 1.56, *p* = .215, *η*^2^*_p_* = .018; helping decision by encounter type by participant race: *F*(1,86) = 0.32, *p* = .572, *η*^2^*_p_* = .004]. However, we noticed that one Black female had responded unusually slowly throughout the task (i.e., her mean response times across all trial types was > 3 *SD*s of the sample’s mean). We therefore re-run the above ANOVA excluding her replies (see Table S10). This time, all main effects reached statistical significance [helping decision: *F*(1,85) = 7.37, *p* = .008, *η*^2^*_p_* = .080; encounter type: *F*(1,85) = 5.44, *p* = .022, *η*^2^*_p_* = .060; and participant race: *F*(1,85) = 4.90, *p* = .029, *η*^2^*_p_* = .055]. But, as before, there were no significant interaction effects [helping decision × participant race: *F*(1,85) = 0.03, *p* = .867, *η*^2^*_p_* < .001; encounter type × participant race: *F*(1,85) = 0.86, *p* = .358, *η*^2^*_p_* = .010; helping decision × encounter type: *F*(1,85) = 2.34, *p* = .130, *η*^2^*_p_* = .027; helping decision by encounter type × participant race: *F*(1,85) = 0.11, *p* = .743, *η*^2^*_p_* = .001]. Finally, correlating participants’ mean response times on the categorization task with their bias on the EPT returned noticeably different results, depending on whether the slow respondent was excluded (see Table S11): Upon excluding her, seemingly significant correlations between Black participants’ mean response times and their cumulative EPT scores disappeared.

Table S9. *Mean response times in ms (and standard deviations) on the categorization task based on helping decision and encounter type or trial type in Experiment 2*

| Person Dyad | Helping Decision | Black Sample | White Sample | *t*-value (df = 86) | *p*-value | Cohen’s *d* |
| --- | --- | --- | --- | --- | --- | --- |
| *By Encounter Type* | | | | | | |
| Same-race | Present | 952 (101) | 989 (106) | 1.66 | .102 | 0.36 |
|  | Absent | 925 (104) | 958 (83) | 1.64 | .105 | 0.35 |
| Cross-race | Present | 945 (113) | 971 (101) | 1.12 | .268 | 0.24 |
|  | Absent | 923 (97) | 953 (87) | 1.53 | .131 | 0.33 |
| *By Trial Type* | | | | | | |
| BBE | Present | 955 (104) | 977 (106) | 1.02 | .311 | 0.22 |
|  | Absent | 928 (102) | 961 (79) | 1.69 | .095 | 0.36 |
| WWE | Present | 950 (105) | 999 (115) | 2.10 | .039 | 0.45 |
|  | Absent | 922 (115) | 955 (100) | 1.46 | .148 | 0.32 |
| BWE | Present | 947 (119) | 979 (106) | 1.33 | .189 | 0.29 |
|  | Absent | 920 (102) | 954 (91) | 1.64 | .105 | 0.35 |
| WBE | Present | 944 (117) | 963 (112) | 0.78 | .438 | 0.17 |
|  | Absent | 924 (102) | 951 (90) | 1.29 | .200 | 0.28 |

Abbreviations: Black-on-Black encounters (BBE), White-on-White encounters (WWE), Black-on-White encounters (BWE), White-on-Black encounters (WBE). *Denotes *p*-values < 0.05 [Bonferroni-corrected for 4 tests (by encounter type) and 8 tests (by trial type)].

Table S10. *Mean response times in ms (and standard deviations) on the categorization task based on helping decision and encounter type or trial type for an outlier-removed sample of participants in Experiment 2*

| Person Dyad | Helping Decision | Black Sample | White Sample | *t*-value (df = 85) | *p*-value | Cohen’s *d* |
| --- | --- | --- | --- | --- | --- | --- |
| *A) By Encounter Type* | | | | | | |
| Same-race | Present | 944 (87) | 989 (106) | 2.14 | .035 | 0.46 |
|  | Absent | 919 (96) | 958 (83) | 2.04 | .044 | 0.44 |
| Cross-race | Present | 935 (92) | 971 (101) | 1.72 | .088 | 0.37 |
|  | Absent | 918 (92) | 953 (87) | 1.82 | .073 | 0.40 |
| *B) By Trial Type* | | | | | | |
| BBE | Present | 947 (90) | 977 (106) | 1.46 | .147 | 0.32 |
|  | Absent | 922 (97) | 961 (79) | 2.00 | .049 | 0.44 |
| WWE | Present | 942 (92) | 999 (115) | 2.56 | .012 | 0.56 |
|  | Absent | 914 (104) | 955 (100) | 1.88 | .063 | 0.41 |
| BWE | Present | 937 (101) | 979 (106) | 1.87 | .065 | 0.41 |
|  | Absent | 914 (93) | 954 (91) | 2.03 | .046 | 0.44 |
| WBE | Present | 934 (94) | 963 (112) | 1.33 | .188 | 0.29 |
|  | Absent | 921 (101) | 951 (90) | 1.46 | .149 | 0.32 |

Abbreviations: Black-on-Black encounters (BBE), White-on-White encounters (WWE), Black-on-White encounters (BWE), White-on-Black encounters (WBE). *Denotes *p*-values < 0.05 [Bonferroni-corrected for 4 tests (by encounter type) and 8 tests (by trial type)].

Table S11. *Correlations (with p-values) between participants’ mean response times on the categorization task and their cumulative evaluative priming task score (EPT score) for the full sample of participants and an outlier-removed sample of participants in Experiment 2*

| Sample | Encounter Type | Helping Decision | Black Sample | White Sample | Total Sample |
| --- | --- | --- | --- | --- | --- |
| Full  (n = 88) | Same-race | Present | .38 (.009)* | .09 (.592) | .20 (.063) |
|  |  | Absent | .38 (.010)* | -.02 (.918) | .17 (.117) |
|  | Cross-race | Present | .43 (.003)* | .13 (.408) | .27 (.013) |
|  |  | Absent | .35 (.017) | -.02 (.893) | .15 (.177) |
| Removed  (n = 87) | Same-race | Present | .06 (.684) | .09 (.592) | .06 (.589) |
|  |  | Absent | .16 (.286) | -.02 (.918) | .05 (.681) |
|  | Cross-race | Present | .08 (.612) | .13 (.408) | .09 (.385) |
|  |  | Absent | .18 (.235) | -.02 (.893) | .05 (.657) |

*Denotes *p*-values < 0.05 (Bonferroni-corrected for 4 tests)

*Whole-Brain Contrasts by Participant Race.* For Black participants (see Table S12), the contrast cross-race > same-race encounters revealed enhanced DMPFC activity, whereas the reverse contrast yielded increased activity in the cerebellum, the posterior cingulate cortex, and the supplementary motor area. For White participants (see Table S13), the contrast cross-race > same-race encounters revealed no suprathreshold activation, whereas the reverse contrast found increased activity in several brain regions, including the right ventral striatum. However, when we examined potential interaction effects by computing the 2 (encounter type: same-race vs. cross-race) × 2 (participant race: Black vs. White) mixed measures ANOVA, no significant suprathreshold activations emerged. As such, the alleged group differences as implied above failed to reach statistical significance when directly compared against each other. To further explore potential group differences that could affect the interpretation of our results, we also checked whether both groups of participants differed in their neural response to each type of encounter relative to baseline (see Table S14): Regardless whether cross-race encounters or same-race encounters were scrutinized, Black participants showed enhanced activity in the right TPJ compared to White participants. The reverse contrast (White > Black) revealed no suprathreshold activation for either contrast. Thus, Black participants engaged the right TPJ more strongly than White participants during encounter processing, irrespective of encounter type

Table S12.

*Black participants: Peak voxel in MNI coordinates and number of voxels for brain regions as identified from the categorization task by exploratory whole-brain analyses at a voxelwise threshold of p < .001, a cluster-size threshold of p < .05 (FDR corrected), and a minimum cluster size of > 10 voxels in Experiment 2*

| Region | Hemi-sphere | Voxels | *T* | *p*(FDR) | *x* | *y* | *z* | |
| --- | --- | --- | --- | --- | --- | --- | --- | --- |
| *Cross-Race Encounters > Same-Race Encounters* | | | | | | | |  |
| Dorsomedial Prefrontal Cortex | Midline | 255 | 5.89 | <.001 | -3 | 59 | 31 | |
| *Same-Race Encounters > Cross-Race Encounters* | | | | | | | |  |
| Cerebellum (extending into the fusiform gyrus) | L | 42 | 5.18  4.18 | .018  .018 | -24  -21 | -67  -73 | -26  -20 | |
| Posterior Cingulate Cortex | Midline | 106 | 4.72 | <.001 | 15 | -28 | 46 | |
| Supplementary Motor Area | Midline | 47 | 4.47 | .016 | 6 | 14 | 46 | |

Table S13.

*White participants: Peak voxel in MNI coordinates and number of voxels for brain regions as identified from the categorization task by exploratory whole-brain analyses at a voxelwise threshold of p < .001, a cluster-size threshold of p < .05 (FDR corrected), and a minimum cluster size of > 10 voxels in Experiment 2.*

| Region | Hemi-sphere | Voxels | *T* | *p*(FDR) | *x* | *y* | *z* | |
| --- | --- | --- | --- | --- | --- | --- | --- | --- |
| *Cross-Race Encounters > Same-Race Encounters* | | | | | | | |  |
| no suprathreshold activation |  |  |  |  |  |  |  | |
| *Same-Race Encounters > Cross-Race Encounters* | | | | | | | |  |
| Cerebellum | L | 32 | 4.91 | .028 | -27 | -43 | -35 | |
| Cuneus | L | 54 | 6.42 | .005 | -24 | -85 | 34 | |
|  | R | 53 | 4.87 | .005 | 9 | -79 | 28 | |
|  | R | 77 | 4.77 | .001 | 24 | -88 | 28 | |
| Fusiform Gyrus | R | 226 | 4.59 | <.001 | 21 | -82 | -14 | |
| Inferior Parietal lobe | R | 29 | 5.31 | .037 | 48 | -34 | 37 | |
| Insula | L | 75 | 5.02 | .001 | -33 | 17 | 7 | |
|  | R | 43 | 4.44 | .011 | 39 | 14 | 1 | |
| Precuneus | Midline | 33 | 4.47 | .028 | -15 | -76 | 52 | |
| Posterior Cingulate Cortex | Midline | 26 | 4.24 | .048 | -15 | -34 | 40 | |
| Supplementary Motor Area | Midline | 185 | 5.43 | <.001 | 12 | 5 | 52 | |
| Ventral Striatum | R | 50 | 4.48 | .006 | 9 | 17 | -2 | |

Table S14.

*Peak voxel in MNI coordinates and number of voxels for brain regions as identified from the categorization task by comparing Black and White participants’ encounter-dependent responses (relative to baseline) with an exploratory whole-brain analyses at a voxelwise threshold of p < .001, a cluster-size threshold of p < .05 (FDR corrected), and a minimum cluster size of > 10 voxels in Experiment 2.*

| Region | Hemi-sphere | Voxels | *T* | *p*(FDR) | *x* | *y* | *z* |
| --- | --- | --- | --- | --- | --- | --- | --- |
| *Cross-Race Encounters vs. Baseline* | | | | | | | |
| *Black > White* | | | | | | | |
| Temporal-Parietal Junction | L | 92 | 4.37 | .002 | -48 | -73 | 37 |
| *White > Black* | | | | | | | |
| no suprathreshold activation | | | | | | | |
| *Same-Race Encounters vs. Baseline* | | | | | | | |
| *Black > White* | | | | | | | |
| Temporal-Parietal Junction | L | 90 | 4.26 | .003 | -48 | -73 | 37 |
| *White > Black* | | | | | | | |
| no suprathreshold activation | | | | | | | |

*ROI Localization Results. Person Perception Network (PPN).* Contrary to our prediction, neither of the ROIs belonging to the PPN showed a main effect of encounter type (see Figure S2 and Tables S15 and S16). For each ROI, the parameter estimates were submitted to a 2 (encounter type: same-race vs. cross-race) × 2 (participant race: Black vs. White) mixed measures ANOVA.


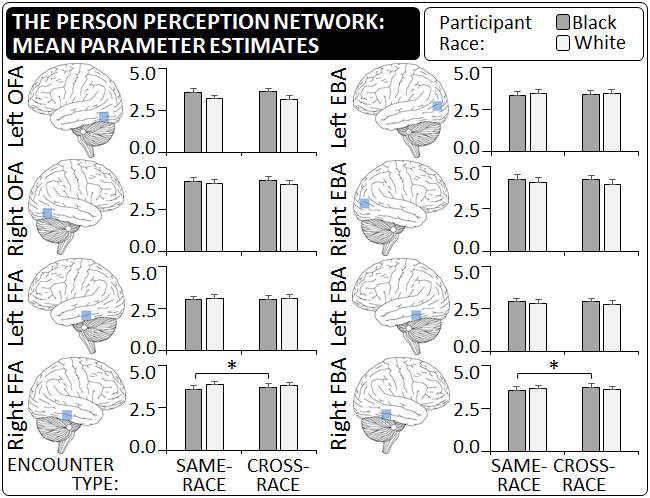


*Figure S2*. In Experiment 2, regions of interest in the person perception network were identified with a separate localizer task and included the occipital face area (OFA), the fusiform face area (FFA), the extrastriate body area (EBA), and the fusiform body area (FBA). The graph shows the regions’ approximate positions and their mean parameter estimates based on participant race and encounter type during the categorization task. Neither region showed a main effect of encounter type, but in two regions (i.e., the right FFA, FBA) significant participant race × encounter type interactions signaled enhanced brain activity for cross-race compared to same-race encounters in Black (but not White) participants. All error bars indicate SEMs.

The right *OFA* displayed no main effect of encounter type [*F*(1,86) = 0.35, *p* = .558, *η*^2^*_p_* = .004] or of participant race [*F*(1,86) = 0.29, *p* = .590, *η*^2^*_p_* = .003] and no encounter type × participant race interaction [*F*(1,86) = 1.87, *p* = .175, *η*^2^*_p_* = .021]. The left *OFA* displayed no main effect of encounter type [*F*(1,81) = 0.00, *p* = .996, *η*^2^*_p_* = .000] or of participant race [*F*(1,81) = 2.16, *p* = .146, *η*^2^*_p_* = .026], and no encounter type × participant race interaction [*F*(1,81) = 1.08, *p* = .302, *η*^2^*_p_* = .013]. The right *FFA* displayed no main effect of encounter type [*F*(1,86) = 0.91, *p* = .343, *η*^2^*_p_* = .010], no main effect of participant race [*F*(1,86) = 0.45, *p* = .507, *η*^2^*_p_* = .005], but a significant encounter type × participant race interaction [*F*(1,86) = 7.04, *p* = .010, *η*^2^*_p_* = .076]. This interaction signalled that in Black participants cross-race encounters elicited enhanced activity in this region compared to same-race encounters [*t*(45) = 2.66, *p* = .011, Cohen’s *d*_z_ = 0.39], whereas in White participants both types of encounters elicited comparable activity [*t*(41) = 1.15, *p* = .255, Cohen’s *d*_z_ = 0.18]. The left *FFA* displayed no main effect of encounter type [*F*(1,83) = 0.36, *p* = .552, *η*^2^*_p_* = .004], no main effect of participant race [*F*(1,83) = 0.01, *p* = .935, *η*^2^*_p_* = .000], and no encounter type × participant race interaction [*F*(1,83) = 0.24, *p* = .623, *η*^2^*_p_* = .003].

The right *EBA* displayed no main effect of encounter type [*F*(1,86) = 3.12, *p* = .081, *η*^2^*_p_* = .035] or of participant race [*F*(1,86) = 0.28, *p* = .596, *η*^2^*_p_* = .003] and no encounter type × participant race interaction [*F*(1,86) = 2.11, *p* = .150, *η*^2^*_p_* = .024]. The left *EBA* displayed no main effect of encounter type [*F*(1,86) = 0.34, *p* = .561, *η*^2^*_p_* = .004], no main effect of participant race [*F*(1,86) = 0.07, *p* = .800, *η*^2^*_p_* = .001], and no encounter type × participant race interaction [*F*(1,86) = 0.05, *p* = .817, *η*^2^*_p_* = .001]. The right *FBA* displayed no main effect of encounter type [*F*(1,86) = 2.48, *p* = .119, *η*^2^*_p_* = .028], no main effect of participant race [*F*(1,86) = 0.00, *p* = .967, *η*^2^*_p_* = .000], but a significant encounter type × participant race interaction [*F*(1,86) = 8.98, *p* = .004, *η*^2^*_p_* = .095]. This interaction signalled that in Black participants cross-race encounters elicited enhanced activity in the right FBA compared to same-race encounters [*t*(45) = 3.43, *p* = .001, Cohen’s *d*_z_ = 0.51]. In White participants, both types of encounters elicited comparable activity in this region [*t*(41) = 0.95, *p* = .348, Cohen’s *d*_z_ = 0.15]. The left *FBA* displayed no main effect of encounter type [*F*(1,83) = 0.02, *p* = .883, *η*^2^*_p_* = .000] or of participant race [*F*(1,83) = 0.27, *p* = .605, *η*^2^*_p_* = .003] and no encounter type × participant race interaction [*F*(1,83) = 1.33, *p* = .252, *η*^2^*_p_* = .016].

Table S15. *Mean parameter estimates (and standard deviations) by encounter type extracted from key nodes of the person perception network in Experiment 2*

| ROI | Person Dyad | Black Sample | White Sample | *t*-value | *p*-value | Cohen’s *d* |
| --- | --- | --- | --- | --- | --- | --- |
| OFA (RH) | Same-race | 4.15 (1.57) | 4.02 (1.51) | 0.40 | .691 | 0.09 |
|  | Cross-race | 4.22 (1.54) | 3.99 (1.61) | 0.67 | .503 | 0.15 |
| OFA (LH) | Same-race | 3.56 (1.32) | 3.16 (1.41) | 1.35 | .183 | 0.30 |
|  | Cross-race | 3.60 (1.31) | 3.12 (1.42) | 1.58 | .119 | 0.35 |
| FFA (RH) | Same-race | 3.58 (1.61) | 3.88 (1.30) | 0.96 | .340 | 0.21 |
|  | Cross-race | 3.71 (1.56) | 3.82 (1.33) | 0.36 | .718 | 0.08 |
| FFA (LH) | Same-race | 3.05 (1.31) | 3.09 (1.60) | 0.14 | .893 | 0.03 |
|  | Cross-race | 3.08 (1.34) | 3.09 (1.66) | 0.03 | .977 | 0.01 |
| EBA (RH) | Same-race | 4.22 (1.81) | 4.06 (1.94) | 0.40 | .691 | 0.09 |
|  | Cross-race | 4.21 (1.85) | 3.95 (1.88) | 0.66 | .510 | 0.14 |
| EBA (LH) | Same-race | 3.34 (1.63) | 3.44 (1.50) | 0.28 | .782 | 0.06 |
|  | Cross-race | 3.37 (1.71) | 3.45 (1.46) | 0.23 | .819 | 0.05 |
| FBA (RH) | Same-race | 3.56 (1.58) | 3.67 (1.27) | 0.36 | .723 | 0.08 |
|  | Cross-race | 3.71 (1.60) | 3.62 (1.24) | 0.27 | .786 | 0.06 |
| FBA  (LH) | Same-race | 2.91 (1.18) | 2.79 (1.54) | 0.39 | .698 | 0.09 |
|  | Cross-race | 2.95 (1.18) | 2.76 (1.60) | 0.64 | .526 | 0.14 |

Abbreviations: occipital face area (OFA), fusiform face area (FFA), extrastriate body are (EBA), fusiform body area (FBA), right hemisphere (RH), left hemisphere (LH), midline structure (mid). *Denotes *p*-values < 0.05 (Bonferroni-corrected for 2 tests per ROI).

Table S16. *Mean parameter estimates (and standard deviations) by trial type extracted from key nodes of the person perception network in Experiment 2*

| ROI | Person Dyad | Black Sample | White Sample | *t*-value | *p*-value | Cohen’s *d* |
| --- | --- | --- | --- | --- | --- | --- |
| OFA (RH) | BBE | 4.28 (1.61) | 4.05 (1.58) | 0.66 | .513 | 0.14 |
|  | WWE | 4.02 (1.56) | 3.99 (1.51) | 0.12 | .906 | 0.02 |
|  | BWE | 4.32 (1.59) | 4.12 (1.69) | 0.56 | .575 | 0.12 |
|  | WBE | 4.12 (1.54) | 3.86 (1.58) | 0.77 | .443 | 0.17 |
| OFA (LH) | BBE | 3.68 (1.43) | 3.18 (1.44) | 1.57 | .122 | 0.35 |
|  | WWE | 3.45 (1.23) | 3.14 (1.41) | 1.07 | .287 | 0.24 |
|  | BWE | 3.69 (1.34) | 3.26 (1.50) | 1.39 | .167 | 0.31 |
|  | WBE | 3.50 (1.31) | 2.99 (1.38) | 1.73 | .088 | 0.38 |
| FFA (RH) | BBE | 3.72 (1.67) | 3.93 (1.38) | 0.66 | .510 | 0.14 |
|  | WWE | 3.44 (1.58) | 3.83 (1.27) | 1.25 | .215 | 0.27 |
|  | BWE | 3.83 (1.64) | 3.94 (1.42) | 0.32 | .750 | 0.07 |
|  | WBE | 3.58 (1.52) | 3.70 (1.30) | 0.40 | .694 | 0.09 |
| FFA (LH) | BBE | 3.18 (1.41) | 3.10 (1.65) | 0.26 | .799 | 0.06 |
|  | WWE | 2.91 (1.26) | 3.08 (1.57) | 0.55 | .582 | 0.12 |
|  | BWE | 3.16 (1.46) | 3.21 (1.75) | 0.15 | .879 | 0.03 |
|  | WBE | 3.01 (1.27) | 2.98 (1.62) | 0.11 | .913 | 0.02 |
| EBA (RH) | BBE | 4.28 (1.85) | 4.05 (1.93) | 0.57 | .570 | 0.12 |
|  | WWE | 4.17 (1.79) | 4.08 (1.99) | 0.22 | .825 | 0.05 |
|  | BWE | 4.28 (1.85) | 4.15 (1.95) | 0.33 | .741 | 0.07 |
|  | WBE | 4.14 (1.87) | 3.75 (1.85) | 0.99 | .327 | 0.21 |
| EBA (LH) | BBE | 3.39 (1.72) | 3.40 (1.49) | 0.02 | .985 | 0.00 |
|  | WWE | 3.30 (1.58) | 3.48 (1.57) | 0.54 | .594 | 0.12 |
|  | BWE | 3.43 (1.73) | 3.62 (1.57) | 0.55 | .585 | 0.12 |
|  | WBE | 3.31 (1.74) | 3.28 (1.39) | 0.11 | .912 | 0.02 |
| FBA (RH) | BBE | 3.68 (1.64) | 3.70 (1.32) | 0.05 | .959 | 0.01 |
|  | WWE | 3.44 (1.55) | 3.64 (1.26) | 0.67 | .507 | 0.15 |
|  | BWE | 3.81 (1.71) | 3.72 (1.31) | 0.27 | .788 | 0.06 |
|  | WBE | 3.60 (1.52) | 3.52 (1.22) | 0.27 | .790 | 0.06 |
| FBA (LH) | BBE | 3.04 (1.31) | 2.79 (1.57) | 0.81 | .418 | 0.18 |
|  | WWE | 2.78 (1.09) | 2.80 (1.54) | 0.08 | .935 | 0.02 |
|  | BWE | 3.02 (1.30) | 2.85 (1.68) | 0.52 | .603 | 0.11 |
|  | WBE | 2.88 (1.12) | 2.67 (1.56) | 0.74 | .459 | 0.16 |

Abbreviations: Black-on-Black encounters (BBE), White-on-White encounters (WWE), Black-on-White encounters (BWE), White-on-Black encounters (WBE), occipital face area (OFA), fusiform face area (FFA), extrastriate body are (EBA), fusiform body area (FBA), right hemisphere (RH), left hemisphere (LH), midline structure (mid). *Denotes *p*-values < 0.05 (Bonferroni-corrected for 4 tests per ROI).

*ROI Localization Results. Mentalizing Network (MTN).* Table S17 shows the extracted mean parameter estimates for all ROIs by encounter type, Table S18 by trial type. For each ROI, the relevant mean parameter estimates were submitted to a 2 (encounter type: same-race vs. cross-race) × 2 (participant race: Black vs. White) mixed measures ANOVA. It was found that the VMPFC displayed a significant main effect of encounter type [*F*(1,86) = 6.76, *p* = .011, *η*^2^*_p_* = .073], signalling stronger activity for cross-race (*M* = 0.52, *SD* = 0.96) than same-race encounters (*M* = 0.38, *SD* = 0.80). The region showed neither a main effect of participant race [*F*(1,86) = 0.50, *p* = .481, *η*^2^*_p_* = .006], nor an encounter type × participant race interaction [*F*(1,86) = 1.06, *p* = .306, *η*^2^*_p_* = .012].

Table S17. *Mean parameter estimates (and standard deviations) by encounter type extracted from key nodes of the mentalizing network in Experiment 2*

| ROI | Encounter Type | Black Sample | White Sample | *t*-value | *p*-value | Cohen’s *d* |
| --- | --- | --- | --- | --- | --- | --- |
| VMPFC (mid) | Same-race | 0.42 (0.86) | 0.34 (0.75) | 0.42 | .673 | 0.09 |
|  | Cross-race | 0.61 (0.95) | 0.43 (0.97) | 0.90 | .372 | 0.19 |
| DMPFC (mid) | Same-race | 0.54 (0.60) | 0.12 (0.78) | 2.91 | .005* | 0.63 |
|  | Cross-race | 0.89 (0.65) | 0.27 (0.71) | 4.26 | <.001* | 0.92 |
| ATL (RH) | Same-race | 0.69 (0.67) | 0.25 (0.56) | 3.35 | .001* | 0.72 |
|  | Cross-race | 0.77 (0.67) | 0.27 (0.61) | 3.62 | .001* | 0.78 |
| ATL (LH) | Same-race | 0.45 (0.59) | 0.15 (0.57) | 2.42 | .018* | 0.52 |
|  | Cross-race | 0.57 (0.67) | 0.20 (0.50) | 2.86 | .005* | 0.62 |
| TPJ (RH) | Same-race | 1.30 (1.24) | 1.27 (1.01) | 0.12 | .902 | 0.03 |
|  | Cross-race | 1.44 (1.32) | 1.36 (0.97) | 0.32 | .751 | 0.07 |
| TPJ (LH) | Same-race | 0.94 (1.31) | 0.61 (0.99) | 1.30 | .198 | 0.28 |
|  | Cross-race | 1.06 (1.39) | 0.74 (1.04) | 1.22 | .227 | 0.26 |
| PrC (mid) | Same-race | 1.41 (0.92) | 0.94 (0.91) | 2.45 | .016* | 0.53 |
|  | Cross-race | 1.64 (1.05) | 1.06 (0.92) | 2.76 | .007* | 0.60 |

Abbreviations: ventromedial prefrontal cortex (VMPFC), dorsomedial prefrontal cortex (DMPFC), anterior temporal lobe (ATL), temporal-parietal junction (TPJ), precuneus (PrC), right hemisphere (RH), left hemisphere (LH), midline structure (mid). *Denotes *p*-values < 0.05 (Bonferroni-corrected for 2 tests per ROI).

The DMPFC, by contrast, displayed two significant main effects [encounter type: *F*(1,86) = 32.94, *p* < .001, *η*^2^*_p_* = .277; participant race: *F*(1,86) = 14.05, *p* < .001, *η*^2^*_p_* = .140] and a significant interaction effect [*F*(1,86) = 4.78, *p* = .032, *η*^2^*_p_* = .053]. The main effect of encounter type signalled stronger activity for cross-race (*M* = 0.59, *SD* = 0.74) than same-race encounters (*M* = 0.34, *SD* = 0.72). The main effect of participant race indicated a stronger response in Black (*M* = 0.72, *SD* = 0.59) than White participants (*M* = 0.19, *SD* = 0.72). The interaction effect revealed a stronger enhancement for cross-race than same-race encounters in Black participants [*t*(45) = 6.05, *p* < .001, Cohen’s *d*_z_ = 0.89] than in White participants [*t*(41) = 2.33, *p* = .025, Cohen’s *d*_z_ = 0.36]. In addition, the right ATL displayed no main effect of encounter type [*F*(1,86) = 2.31, *p* = .132, *η*^2^*_p_* = .026], but a significant main effect of participant race [*F*(1,86) = 12.88, *p* = .001, *η*^2^*_p_* = .130], and no interaction effect [*F*(1,86) = 0.61, *p* = .438, *η*^2^*_p_* = .007]. The main effect of participant race indicated a stronger response in Black (*M* = 0.73, *SD* = 0.65) than White participants (*M* = 0.26, *SD* = 0.65). By contrast, the left ATL displayed two significant main effects [encounter type: *F*(1,85) = 7.84, *p* = .006, *η*^2^*_p_* = .084; participant race: *F*(1,85) = 7.47, *p* = .008, *η*^2^*_p_* = .081], but no interaction effect [*F*(1,85) = 1.05, *p* = .308, *η*^2^*_p_* = .012]. The main effect of encounter type signalled stronger activity for cross-race (*M* = 0.40, *SD* = 0.62) than same-race encounters (*M* = 0.31, *SD* = 0.60). The main effect of participant race indicated a stronger response in Black (*M* = 0.51, *SD* = 0.62) than White participants (*M* = 0.18, *SD* = 0.51). The right TPJ displayed a significant main effect of encounter type [*F*(1,86) = 10.74, *p* = .002, *η*^2^*_p_* = .111], no main effect of participant race [*F*(1,86) = 0.05, *p* = .823, *η*^2^*_p_* = .001], and no interaction effect [*F*(1,86) = 0.47, *p* = .494, *η*^2^*_p_* = .005]. The main effect of encounter type signalled stronger activity for cross-race (*M* = 1.40, *SD* = 1.16) than same-race encounters (*M* = 1.29, *SD* = 1.13). Similarly, the left TPJ displayed a significant main effect of encounter type [*F*(1,86) = 8.94, *p* = .004, *η*^2^*_p_* = .094], no main effect of participant race [*F*(1,86) = 1.62, *p* = .207, *η*^2^*_p_* = .018], and no interaction effect [*F*(1,86) = 0.00, *p* = .966, *η*^2^*_p_* = .000]. The main effect of encounter type signalled stronger activity for cross-race (*M* = 0.91, *SD* = 1.24) than same-race encounters (*M* = 0.78, *SD* = 1.17). Last but not least, the PrC displayed two significant main effects [encounter type [*F*(1,86) = 12.04, *p* = .001, *η*^2^*_p_* = .123; participant race: *F*(1,86) = 7.24, *p* = .009, *η*^2^*_p_* = .078], but no interaction effect [*F*(1,86) = 1.07, *p* = .303, *η*^2^*_p_* = .012]. The main effect of encounter type signalled stronger activity for cross-race (*M* = 1.36, *SD* = 1.03) than same-race encounters (*M* = 1.19, *SD* = 0.94). The main effect of participant race indicated a stronger response in Black (*M* = 1.53, *SD* = 0.95) than White participants (*M* = 1.00, *SD* = 0.89).

Table S18. *Mean parameter estimates (and standard deviations) by trial type extracted from key nodes of the mentalizing network in Experiment 2*

| ROI | Person Dyad | Black Sample | White Sample | *t*-value | *p*-value | Cohen’s *d* |
| --- | --- | --- | --- | --- | --- | --- |
| VMPFC (mid) | BBE | 0.56 (0.98) | 0.41 (0.85) | 0.78 | .439 | 0.17 |
|  | WWE | 0.27 (0.89) | 0.28 (0.76) | 0.04 | .968 | 0.01 |
|  | BWE | 0.60 (1.05) | 0.46 (1.10) | 0.63 | .531 | 0.13 |
|  | WBE | 0.62 (0.91) | 0.40 (0.92) | 1.15 | .254 | 0.25 |
| DMPFC (mid) | BBE | 0.73 (0.68) | 0.22 (0.92) | 2.96 | .004* | 0.64 |
|  | WWE | 0.36 (0.65) | 0.01 (0.78) | 2.29 | .024 | 0.50 |
|  | BWE | 0.86 (0.73) | 0.24 (0.79) | 3.86 | <.001* | 0.83 |
|  | WBE | 0.92 (0.72) | 0.30 (0.74) | 3.94 | <.001* | 0.85 |
| ATL (RH) | BBE | 0.74 (0.74) | 0.27 (0.63) | 3.20 | .002* | 0.69 |
|  | WWE | 0.64 (0.65) | 0.23 (0.58) | 3.15 | .002* | 0.67 |
|  | BWE | 0.87 (0.71) | 0.30 (0.79) | 3.59 | .001* | 0.77 |
|  | WBE | 0.66 (0.72) | 0.25 (0.50) | 3.13 | .002* | 0.67 |
| ATL (LH) | BBE | 0.52 (0.66) | 0.20 (0.63) | 2.28 | .025 | 0.50 |
|  | WWE | 0.38 (0.61) | 0.09 (0.58) | 2.24 | .028 | 0.49 |
|  | BWE | 0.55 (0.77) | 0.17 (0.58) | 2.59 | .011* | 0.56 |
|  | WBE | 0.59 (0.65) | 0.24 (0.50) | 2.83 | .006* | 0.61 |
| TPJ (RH) | BBE | 1.35 (1.35) | 1.31 (1.05) | 0.18 | .861 | 0.04 |
|  | WWE | 1.24 (1.23) | 1.23 (1.03) | 0.06 | .952 | 0.01 |
|  | BWE | 1.45 (1.47) | 1.34 (1.02) | 0.41 | .685 | 0.09 |
|  | WBE | 1.43 (1.22) | 1.38 (1.00) | 0.20 | .843 | 0.04 |
| TPJ (LH) | BBE | 1.03 (1.38) | 0.65 (1.07) | 1.42 | .159 | 0.31 |
|  | WWE | 0.84 (1.30) | 0.57 (0.97) | 1.09 | .277 | 0.24 |
|  | BWE | 1.12 (1.52) | 0.73 (1.17) | 1.32 | .189 | 0.29 |
|  | WBE | 1.00 (1.34) | 0.74 (0.98) | 1.01 | .315 | 0.22 |
| PrC (mid) | BBE | 1.62 (1.06) | 1.04 (0.96) | 2.68 | .009* | 0.58 |
|  | WWE | 1.21 (0.89) | 0.83 (0.94) | 1.94 | .056 | 0.42 |
|  | BWE | 1.63 (1.05) | 1.07 (1.04) | 2.53 | .013 | 0.54 |
|  | WBE | 1.65 (1.16) | 1.05 (0.93) | 2.66 | .009* | 0.57 |

Abbreviations: ventromedial prefrontal cortex (VMPFC), dorsomedial prefrontal cortex (DMPFC), anterior temporal lobe (ATL), temporal-parietal junction (TPJ), precuneus (PrC), right hemisphere (RH), left hemisphere (LH), midline structure (mid). *Denotes *p*-values < 0.05 (Bonferroni-corrected for 4 tests per ROI).

*Psychophysiological Interaction (PPI) Results by Participant Race.* Significant voxels in the left VS were identified for 43 Black participants (mean peak voxel MNI coordinates: x = -12, y = 11, z = -10) and for 37 White participants (mean peak voxel MNI coordinates: x = -15, y = 11, z = -8). Similarly, significant voxels in the right VS were identified for 42 Black participants (mean peak voxel MNI coordinates: x = 14, y = 10, z = -9) and for 37 White participants (mean peak voxel MNI coordinates: x = 17, y = 10, z = -6). When the left VS was specified as the PPI’s seed region in Black participants only, enhanced functional connectivity during same-race relative to cross-race encounters was found with the VMPFC, right TPJ, right cerebellum, and the right middle occipital gyrus (see Table S19). For White participants, no suprathreshold activations emerged from this analysis, but enhanced connectivity with the VMPFC reached marginal statistical significance (see Table S20). By contrast, when specifying the right VS as the PPI’s seed region, suprathreshold activations emerged neither for Black, nor White participants.

Table S19. *Peak voxel(s) in MNI coordinates and number of voxels for brain regions showing systematically reduced or enhanced functional connectivity with the left VS in the categorization task for Black participants as identified by psychophysiological interaction (PPI) analyses at a voxelwise threshold of p < .001, a cluster-size threshold of p < .05 (FDR corrected), and a minimum cluster size of > 10 voxels in Experiment 2.*

| Region | Hemi-sphere | Voxels | *T* | *p*(FDR) | *x* | *y* | *z* |
| --- | --- | --- | --- | --- | --- | --- | --- |
| *Same-Race Encounters > Cross-Race Encounters* | | | | | | | |
| Cerebellum | R | 103 | 4.96 | <0.001 | 36 | -64 | -29 |
| Middle Occipital Gyrus | R | 161 | 5.53 | <0.001 | 27 | -97 | 16 |
|  |  |  | 4.82 | <0.001 | 30 | -94 | 4 |
| Temporoparietal Junction | R | 84 | 4.83 | 0.001 | 54 | -64 | 16 |
| Ventromedial Prefrontal Cortex | Midline | 47 | 5.19 | 0.018 | 9 | 59 | -8 |
|  |  |  | 4.33 | 0.018 | -9 | 59 | -8 |
| *Cross-Race Encounters > Same-Race Encounters* | | | | | | | |
| no suprathreshold activation | | | | | | | |

Table S20. *Peak voxel in MNI coordinates and number of voxels for brain regions showing systematically reduced or enhanced functional connectivity with the left VS in the categorization task for White participants as identified by psychophysiological interaction (PPI) analyses at a voxelwise threshold of p < .001, a cluster-size threshold of p < .05 (FDR corrected), and a minimum cluster size of > 10 voxels in Experiment 2.*

| Region | Hemi-sphere | Voxels | *T* | *p*(FDR) | *x* | *y* | *z* |
| --- | --- | --- | --- | --- | --- | --- | --- |
| *Same-Race Encounters > Cross-Race Encounters* | | | | | | | |
| Ventromedial Prefrontal Cortex | Midline | 27 | 4.95 | 0.074 | 9 | 59 | -8 |
| *Cross-Race Encounters > Same-Race Encounters* | | | | | | | |
| no suprathreshold activation | | | | | | | |

*Brain-Behavior Correlations by Participant Race.* Exploratory whole-brain correlations for Black participants revealed no suprathreshold activations, regardless whether positive or negative correlations were examined. For White participants, there were no suprathreshold activations for positive correlations, but several brain regions showed negative correlations, among them the bilateral posterior insula and the nucleus caudate (see Table S19). Additional ROI-based correlations between Black and White participants’ brain activity in the left and right VS and their EPT bias returned several marginally significant findings (see Table S21 and Figure S3): For Black participants, their EPT bias in favor of same-race encounters was larger, the less their VS showed activity increases for same-race relative to cross-race encounters on the categorization task. By contrast, for White participants, their EPT bias was larger, the more their VS showed activity increases for same-race relative to cross-race encounters. Tables S23 and S24, finally, list the non-significant correlations for all ROIs in the mentalizing network and the person perception network.

Table S21. *Peak voxel in MNI coordinates and number of voxels for brain regions as identified from the categorization task by correlating White participants’ neural differences for cross-race and same-race encounters [computed as cross-race encounters – same-race encounters] with their bias on the evaluative priming task (EPT) at a voxelwise threshold of p < .001, a cluster-size threshold of p < .05 (FDR corrected), and a minimum cluster size of > 10 voxels in Experiment 2.*

| Region | Hemi-sphere | Voxels | *T* | *p*(FDR) | *x* | *y* | *z* |
| --- | --- | --- | --- | --- | --- | --- | --- |
| Cerebellum | Midline | 85 | 4.98 | .001 | 3 | -49 | 1 |
| Insula (extending into the superior temporal sulcus) | Left | 52 | 5.15 | .013 | -54 | 8 | -8 |
|  | Right | 88 | 5.13  4.47 | .001  .001 | 54  48 | 11  14 | -8  -11 |
| Nucleus Caudate | Midline | 35 | 4.43 | .045 | 12 | -1 | 16 |
| Postcentral Gyrus | Right | 38 | 4.41 | .041 | 54 | -13 | 16 |

Table S22. *Correlations (with p-values)between participants’ bias in favor of same-race encounters on the evaluative priming task and their neural activity difference (cross-race – same race encounters) in the ventral striatum in Experiment 2*

| Region of Interest | Black Sample | White Sample | Total Sample |
| --- | --- | --- | --- |
| Ventral Striatum (RH) | .27 (.072) | -.35 (.024)* | -.07 (.516) |
| Ventral Striatum (LH) | .30 (.041) | -.29 (.067) | -.05 (.668) |

*Denotes *p*-values < 0.05 (Bonferroni-corrected for 2 tests per sample).

Table S23. *Correlations (with p-values) between participants’ bias on the evaluative priming task and their neural activity difference (cross-race – same race encounters) in ROIs of the mentalizing network in Experiment 2*

| Region of Interest | Black Sample | White Sample | Total Sample |
| --- | --- | --- | --- |
| VMPFC (mid) | -.10 (.512) | -.22 (.160) | -.15 (.163) |
| DMPFC (mid) | -.04 (.805) | -.17 (.288) | -.08 (.454) |
| ATL (RH) | .06 (.680) | -.34 (.030) | -.14 (.182) |
| ATL (LH) | -.05 (.747) | -.31 (.047) | -.18 (.100) |
| TPJ (RH) | -.06 (.686) | -.03 (.848) | -.04 (.740) |
| TPJ (LH) | .12 (.431) | -.22 (.168) | -.06 (.606) |
| PrC (mid) | -.01 (.949) | .03 (.832) | .02 (.840) |

Abbreviations: ventromedial prefrontal cortex (VMPFC), dorsomedial prefrontal cortex (DMPFC), anterior temporal lobe (ATL), temporal-parietal junction (TPJ), precuneus (PrC), right hemisphere (RH), left hemisphere (LH), midline structure (mid). *Denotes *p*-values < 0.05 (Bonferroni-corrected for 7 tests per sample).

Table S24. *Correlations (with p-values) between participants’ bias in favor of same-race encounters on the evaluative priming task and their neural activity difference (cross-race – same race encounters) in ROIs of the person perception network in Experiment 2*

| Region of Interest | Black Sample | White Sample | Total Sample |
| --- | --- | --- | --- |
| Right OFA | .338 (.021) | -.251 (.110) | .011 (.918) |
| Left OFA | .018 (.908) | -.222 (.175) | -.098 (.377) |
| Right FFA | .078 (.604) | -.244 (.119) | -.059 (.587) |
| Left FFA | .150 (.327) | -.385 (.014) | -.161 (.141) |
| Right EBA | .114 (.451) | -.304 (.051) | -.086 (.426) |
| Left EBA | .067 (.657) | -.406 (.008) | -.195 (.068) |
| Right FBA | .257 (.085) | -.050 (.751) | .119 (.269) |
| Left FBA | .091 (.564) | -.182 (.250) | -.056 (.611) |

Abbreviations: occipital face area (OFA), fusiform face area (FFA), extrastriate body area (EBA), fusiform body area (FBA), right hemisphere (RH), left hemisphere (LH). *Denotes *p*-values < 0.05 (Bonferroni-corrected for 8 tests per sample).

**
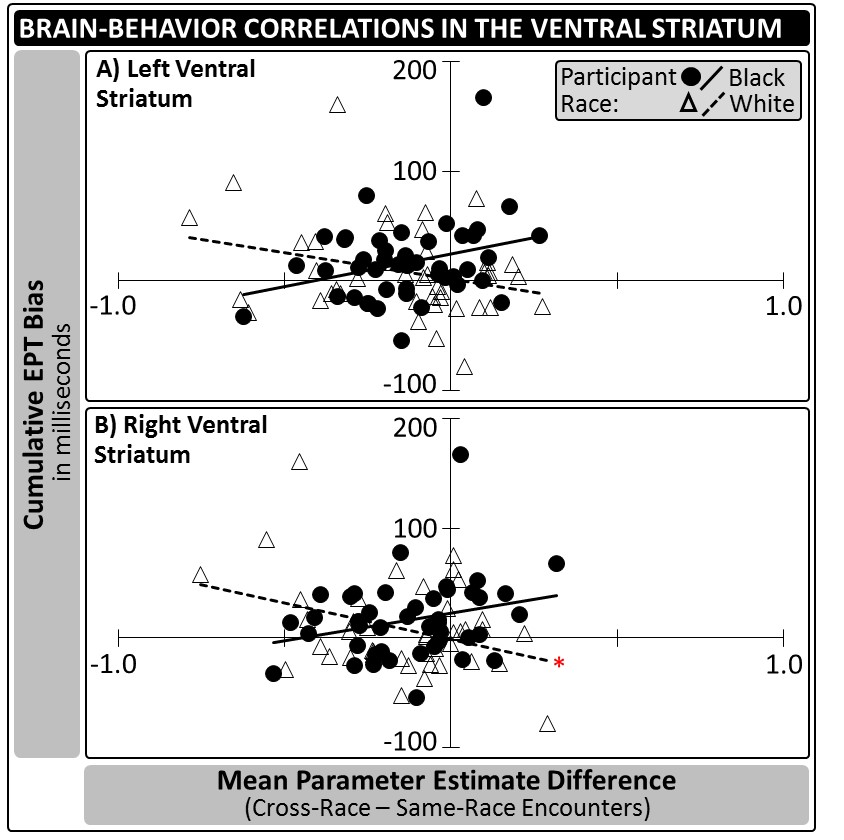
**

*Figure S3*. Correlations between participants’ neural differentiation for cross-race and same-race encounters in the left and right ventral striatum with their bias on the evaluative priming task in Study 2 displayed by participants’ race. *Denotes *p*-values < 0.05 (Bonferroni-corrected).

**References**

Fazio, R. H., Jackson, J. R., Dunton, B. C., & Williams, C. J. (1995) Variability in automatic activation as an unobtrusive measure of racial attitudes: A bona fide pipeline. *Journal of Personality and Social Psychology*, *69*, 1013-1027.

Islam, M. R., & Hewstone, M. (1993). Dimensions of contact as predictors of intergroup anxiety, perceived out-group variability, and out-group attitude: An integrative model. *Personality and Social Psychology Bulletin*, 19, 700-710.

Leach, C. W., van Zomeren, M., Zebel, S., Vliek, M. L. W., Pennekamp, S. F., Doosje, B. et al. (2008). Group-level self-definition and self-investment: A hierarchical (multicomponent) model of in-group identification. *Journal of Personality and Social Psychology*, 95, 144-165.

Pratto, F., Ḉidam, A., Stewart, A. L., Zeineddine, F. B., Aranda, M., Aiello, A. et al (2013). Social dominance in context and in individuals: Contextual moderation of robust effects of social dominance orientation in 15 languages and 20 countries. *Social Psychology and Personality Science*, *4*, 587-599.

Voci, A., & Hewstone, M. (2003). Intergroup contact and prejudice toward immigrants in Italy: The mediational role of anxiety and the moderational role of group salience. *Group Processes and Intergroup Relations*, *6*, 37-54.

Williams, M. J., & Eberhardt, J. L. (2008). Biological conceptions of race and the motivation to cross racial boundaries*. Journal of Personality and Social Psychology*, *4*, 1033-1047.

Wolsko, C., Park, B., & Judd, M. (2006). Considering the tower of Babel: Correlates of assimilation and multiculturalism among ethnic minority and majority groups in the United States. *Social Justice Research*, *19*, 277-306.
